# Hydrogel Microspheres as Versatile Platforms for Biomedical Research: Design, Properties, and Applications

**DOI:** 10.1002/mco2.70423

**Published:** 2025-10-09

**Authors:** Meng Yang, Yuanyuan Shi, Feng Wang, Xin Zhang, Jiayi Shao, Fan Yang, Hao Sun, Chong Zhang, Zheng Zhou, Jianyong Huang, Pengyu Lv, Patrick Shu‐Hang Yung, Jin Cheng, Hong‐Jie Huang, Jian‐Quan Wang

**Affiliations:** ^1^ Department of Sports Medicine Beijing Key Laboratory of Sports Injuries Peking University Third Hospital Beijing China; ^2^ Institute of Sports Medicine of Peking University Beijing China; ^3^ Shenzhen Institute of Advanced Technology Chinese Academy of Sciences Shenzhen Guangdong China; ^4^ College of Engineering Peking University Beijing China; ^5^ Institute For Tissue Engineering and Regenerative Medicine School of Biomedical Sciences Faculty of Medicine The Chinese University of Hong Kong Shatin Hong Kong S. A. R., China

**Keywords:** biomaterials, hydrogel microspheres, tissue engineering

## Abstract

Hydrogel microspheres (HMs) are versatile biomaterials with biocompatibility and controlled release properties, widely applied in drug delivery, cell carriers, and tissue engineering. Their tunable material compositions (natural, synthetic, or composite polymers) and diverse fabrication techniques (e.g., microfluidics, electrohydrodynamic spraying) allow precise regulation of size, morphology, and functionality, supporting applications from musculoskeletal repair to dermatological therapy. Despite rapid advancements, a comprehensive understanding of HM design, manufacturing, and biomedical applications is still lacking, as existing reviews mainly focus on single fields or specific scenarios. This review systematically summarizes HMs construction strategies (material selection and property modulation), fabrication technologies (batch emulsion, microfluidic chips, and emerging Artificial Intelligence (AI)‐assisted methods), and multifunctional applications (drug and cell delivery, nanoparticle integration, and lubrication modification). It highlights the cross‐system therapeutic potential of HMs and discusses challenges in clinical translation. By integrating these aspects, this review aims to bridge the gap between material design and clinical translation, providing researchers with an overview from basic research to clinical application, while exploring approaches to cross‐system synergistic therapy and addressing bottlenecks in clinical translation.

## Graphical abstract

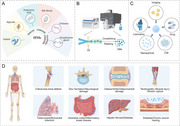

## 1 Introduction

In recent years, minimally invasive approaches for treating tissue damage have garnered significant attention. Hydrogel microspheres (HMs), due to their unique physical and chemical properties, have become crucial in bioactive protein, drug, and cell delivery [1, 2]. Hydrogels, as cross‐linked networks of hydrophilic polymers, can absorb large volumes of water while maintaining structural integrity [3]. When produced in microsphere form, hydrogels combine high surface area, controllable porosity, and tunable mechanical properties, making them excellent candidates for precise material performance regulation [1].

Researchers have leveraged microfluidic technologies to fabricate monodisperse microspheres [4, 5]. In particular, soft lithography based on polydimethylsiloxane (PDMS) laid the foundation for modern HMs technologies [6], enabling precise control over microsphere size, morphology, and functionality. Compared with other particulate systems, such as liposomes [7, 8] and nanoparticles [9, 10], HMs exhibit distinct advantages: they allow higher loading capacity for large biomolecules (e.g., growth factors and cells), provide superior mechanical stability in vivo, and offer customizable degradation properties. For instance, unlike liposomes (which are prone to rapid leakage) or nanoparticles (which are limited in cell encapsulation due to their small size), HMs can codeliver cells, drugs, and gene‐editing tools, making them ideal for complex therapeutic regimens [1, 11]. Clinically, this translates into tangible benefits. For example, HMs loaded with chondrogenic drugs and mesenchymal stem cells (MSCs) can be injected into damaged joints, providing a minimally invasive solution for conditions such as osteoarthritis (OA) [12, 13, 14].

A key advantage of HMs over bulk hydrogels lies in their enhanced oxygen and nutrient transport, attributed to their high surface area‐to‐volume ratio. This facilitates rapid cell proliferation and prevents the core necrosis often observed in large bulk scaffolds [15, 16]. Despite their broad potential, several challenges remain in HM development. In recent years, researchers have optimized synthesis processes to improve loading capacity and release performance, developed novel polymer materials to enhance biological functionality, targeting ability, and stability, and explored key aspects such as biodegradability, long‐term stability, and lubricating capacity for in vivo applications. However, currently published review articles show significant limitations [17, 18, 19, 20]. Most focus on a single field or analyze only specific application scenarios, often lacking systematic analysis of the mechanistic links between material selection and microsphere performance. Furthermore, summaries of clinical translation remain largely confined to laboratory‐based outcomes and fail to fully address critical challenges such as large‐scale production and long‐term in vivo safety.

**FIGURE 1:**
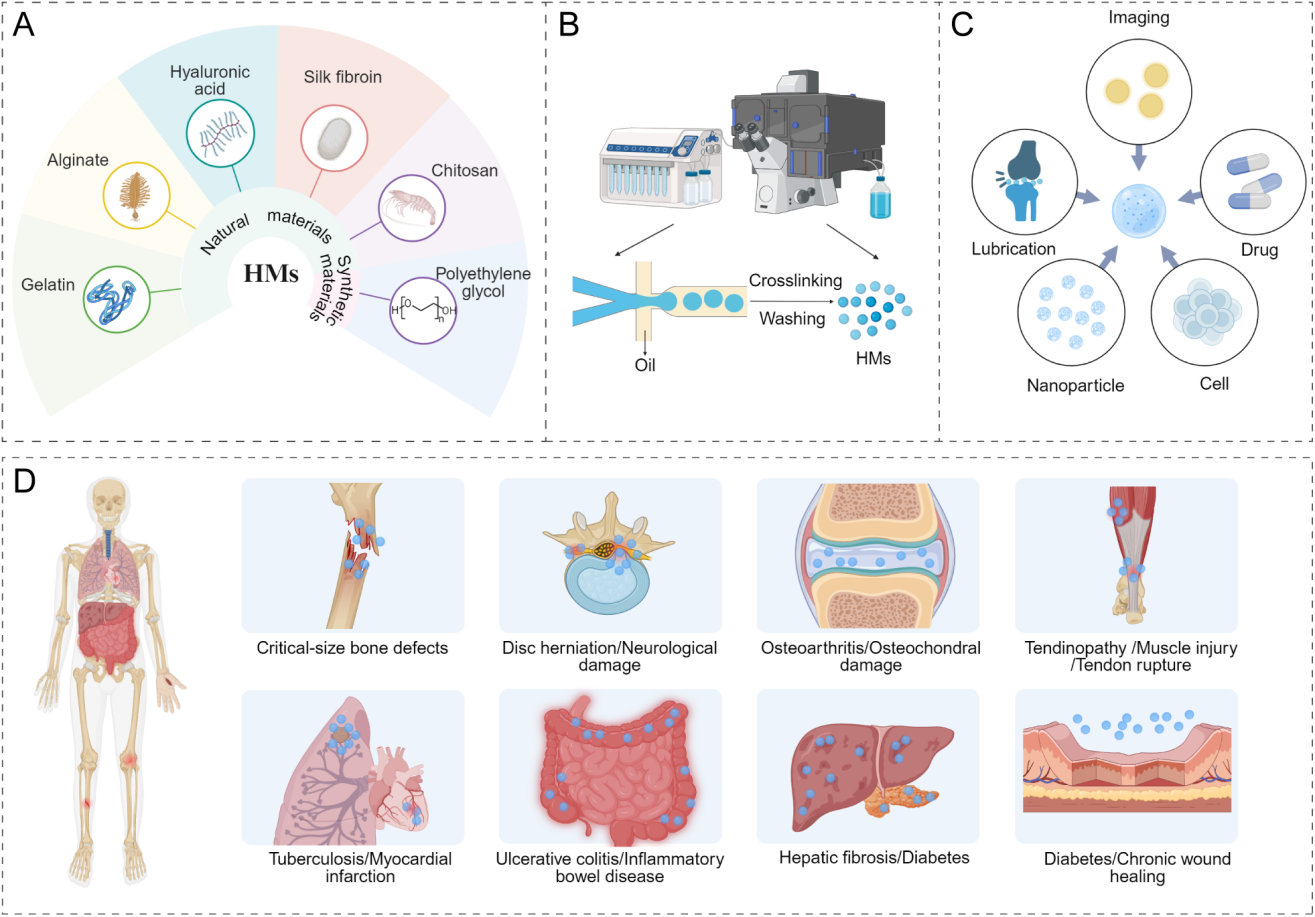
Schematic diagram illustrating the comprehensive landscape of hydrogel microspheres (HMs). (A) The components of HMs. (B) Preparation technologies employed in fabricating HMs. (C) The diverse applications of HMs across different fields. (D) The therapeutic applications of HMs in the treatment of various systemic diseases, demonstrating their potential in clinical interventions.

This review aims to provide a comprehensive overview of recent advances in HMs technology, as illustrated in Figure 1. It explores the advantages of using different biomaterials to form HMs in regenerative medicine. Specifically, we elaborate on natural, synthetic, and composite materials used for HM preparation, with emphasis on how material selection influences microsphere properties. We also compare HMs fabrication techniques, including batch emulsion, photolithography, microfluidic chip technology, and electrohydrodynamic (EHD) spraying, as well as emerging methods such as droplet digital microfluidics. In addition, we describe the multifunctional applications of HMs in drug delivery, cell therapy, and diagnostics, with particular focus on synergistic strategies involving multifunctional composite systems. We summarize HMs applications across a wide range of systemic diseases, including musculoskeletal, neurological, dermatological, cardiovascular, respiratory, and gastrointestinal conditions. Finally, we review the current status of clinical translation, explore the potential for cross‐system applications, and discuss the limitations of existing HMs while outlining future directions for their development in regenerative medicine.

## 2 Composition of HM Structures

Most biomaterials used for tissue regeneration consist of either natural or synthetic organic compounds. Selecting the appropriate biomaterial is crucial for effective tissue regeneration [21]. Regenerative repair materials often demonstrate excellent bioactivity, and specific chemical groups on their surfaces can influence key cellular processes, including proliferation, migration, adhesion, and differentiation.

Hydrogels are highly hydrophilic porous polymer materials, generally categorized as natural or synthetic. Natural hydrogels exhibit excellent biocompatibility but suffer from poor mechanical properties and high swelling rates. In contrast, synthetic hydrogels offer favorable physical and chemical properties, allowing their mechanical strength and degradation rates to be tailored for specific applications [22, 23].

Careful consideration of both biocompatibility and functional characteristics of materials can significantly enhance HM applications in tissue engineering. Typically, HMs are composed of various materials that can be categorized based on their origin, properties, and applications. Commonly used materials include gelatin, alginate, hyaluronic acid (HA), silk fibroin, chitosan (CS), and polyethylene glycol (PEG). The sources, advantages, and disadvantages of these materials are summarized in Table 1.

**TABLE 1:** Comparison of HMs biomaterials.

Hydrogel material	Sources	Advantages	Disadvantages	References
Gelatin	The skin, bone and connective tissue of animals	Containing cell adhesion sites RGD, exhibiting structure and mechanical properties similar to natural tissues, promoting cell proliferation and differentiation	Poor thermal stability and low mechanical strength	[24, 25]
Alginate	Brown algae	Biocompatibility, ionic crosslinking ability, and biodegradability	Excessive swelling properties and low cell adhesion	[26, 27, 28]
Hyaluronic acid	Animal tissue extraction or microbial fermentation	Biocompatibility, high hydrophilicity, and lubricating function	Poor mechanical properties and rapid degradation	[29, 30]
Silk fibroin	Silk	Excellent mechanical properties, thermal stability, and ease of surface modification.	Poor water solubility, lack of RGD sequences, which often restricts cell diffusion and growth	[31, 32, 33, 34]
Chitosan	Deacetylated chitosan product	Biocompatibility, antibacterial properties, and self‐healing performance	Poor water solubility and high pH sensitivity	[35, 36, 37]
Polyethylene glycol	Artificial synthesis	Excellent water solubility, wide particle size distribution, controllable arm length molecular weight, and ease of modification	Poor cell adhesion, lack of bioactivity, and potential immunogenicity	[38, 39, 40]
Composite	Composite	Complex and diverse functions	The production and preparation processes are characterized by intricate complexity	[41, 42, 43, 44]

### 2.1 Natural Polymer Materials

#### 2.1.1 Gelatin

Gelatin is a denatured protein obtained from collagen hydrolysis, with advantages such as low cost, good biocompatibility, and biodegradability [24]. Gelatin retains several bioactive sites from collagen, including the Arg–Gly–Asp (RGD) sequence, which promotes cell adhesion, and sequences that support the enzymatic activity of matrix metalloproteinases (MMPs). These properties play a significant role in tissue repair and regeneration. However, gelatin lacks a stable triple‐helix structure, resulting in poor mechanical strength in gelatin hydrogels [25]. Notably, gelatin contains various reactive groups, such as amino (‐NH_2_), hydroxyl (‐OH), and carboxyl (‐COOH), providing abundant modification sites. Consequently, coupling, crosslinking, and grafting methods can enhance the functionality of gelatin, thereby increasing its practical value. Among these approaches, methacrylated gelatin is the most widely used. This modification introduces methacrylic acid groups onto the amino side chains of gelatin, preserving its fundamental properties while conferring photopolymerization capabilities under ultraviolet and visible light. Gelatin methacryloyl (GelMA) hydrogels can undergo crosslinking and solidification with other polymers in the presence of photoinitiators and specific wavelength illumination, significantly enhancing their mechanical and physical properties [45].

#### 2.1.2 Alginate

Alginate is a natural water‐soluble linear polysaccharide polymer known for its ionic crosslinking ability, environmental sustainability, biocompatibility, and biodegradability [26]. Its abundant surface functional groups and electronegativity make it widely used as an adsorbent for cationic metals. Sodium alginate (SA) consists of two repeating units, 1,4‐β‐d‐mannuronic acid and α‐l‐guluronic acid, interconnected by β‐1,4‐glycosidic bonds. SA molecules form hydrogels by crosslinking with cations such as Ca^2^⁺, Mg^2^⁺, Cu^2^⁺, Pb^2^⁺, and Cd^2^⁺ [27]. In addition, alginate crosslinked with Ca^2^⁺ or Ba^2^⁺ ions can form hydrogels and encapsulate cells under mild conditions [28]. However, natural alginate may contain impurities such as polyphenols, proteins, and lipopolysaccharides, which reduce its biocompatibility. Its poor mechanical properties, low cell adhesion, and excessive swelling due to ionic charges further limit the utility of alginate hydrogels.

#### 2.1.3 Hyaluronic Acid

HA, a linear polysaccharide composed of alternating β‐1,4‐d‐glucuronic acid and β‐1,3‐N‐acetyl‐d‐glucosamine disaccharide units, is abundant in the human body [29]. As a key component of the extracellular matrix (ECM), its structure and biological properties enable roles in cellular signaling, wound healing, morphogenesis, and tissue organization. Animal tissue extraction and microbial fermentation are the most common methods for producing HA. However, HA obtained through these methods has disadvantages [46], including poor stability, sensitivity to hyaluronidases and free radicals, easy degradation, short in vivo retention time, and lack of mechanical strength in aqueous systems. To address these limitations, crosslinking reactions utilizing HA's four functional groups, acetylamide, carboxyl, hydroxyl, and aldehyde, have become a major focus of research [30]. For example, hydrazine compounds such as adipic acid dihydrazide enhance mechanical strength and stability when used as crosslinkers, while glycidyl methacrylate modification generates photo‐crosslinked hydrogels with improved mechanical properties and slower degradation [47].

#### 2.1.4 Silk Fibroin

Silk fibroin, a natural fibrous protein comprising 70–80% of silk, contains 18 amino acids, with glycine, alanine, and serine accounting for over 80%. It offers excellent mechanical properties, biocompatibility, biodegradability, and structural adjustability compared with other natural biopolymers [31]. With high molecular weight and complex intermolecular interactions, its unique β‐sheet structure forms tight hydrogen bonds between antiparallel chains, while hydrophobic regions interspersed with hydrophilic areas enhance fiber strength and elasticity [32]. However, its high crystallinity hinders water solubility, leading to uneven gel formation. During silk degumming and sericin reconstruction in aqueous solutions, many factors can affect sericin stability [33]. Additionally, materials composed of pure silk fibroin show rapid enzymatic degradation [34]. Importantly, silk fibroin's amino acid chains and side chains contain abundant active groups (hydroxyl, phenolic hydroxyl, carboxyl, amino), enabling chemical modification or composite formation for diverse applications.

#### 2.1.5 Chitosan

CS, a cationic polysaccharide derived from chitin deacetylation, contains free amino and hydroxyl groups, enabling extensive chemical modification and crosslinking [35, 48]. Its positive charge facilitates interactions with anionic bacterial membranes, causing intracellular leakage and conferring antibacterial activity. CS also exhibits excellent biocompatibility, biodegradability, nonimmunogenicity, permeability, reliable adhesion, hemostatic properties, antibacterial activity, and low toxicity, making it widely applied in wound dressings that promote healing [49]. However, its macromolecular structure and high amino content limit solubility. Amino group‐based modifications are often employed to form highly crosslinked hydrogel networks. Notably, amino groups react rapidly with aldehydes to form reversible Schiff bases, which are key for hydrogel self‐healing [36]. CS hydrogels exhibit high stiffness, with Young's modulus ranging from several to tens of MPa [37]. Due to the lack of adhesive sequences, they do not promote cell adhesion; however, incorporation of RGD sequences can enhance this property.

### 2.2 Synthetic Polymer Materials

PEG, a polymer of repeating ethylene glycol units with a molecular weight range from 200 to over 8000, is United States Food and Drug Administration (US FDA) approved and widely used because of its unique physicochemical and biological properties. The multifunctionality of the PEG molecular structure, along with its excellent biocompatibility, low immunogenicity, and tunable mechanical and structural characteristics, makes PEG‐based hydrogels one of the most widely applied matrices for drug delivery and scaffolds in tissue engineering. Crosslinking methods include click chemistry, free radical polymerization, enzyme‐catalyzed crosslinking, and chemical reactions between PEG chain end groups and various functional groups. Chemically crosslinked gels exhibit stable structures with tunable properties in water content, rheology, diffusion, and in vivo stability [38]. Notably, increased pharmaceutical use of PEG has raised concerns about immunogenicity, as it can induce anti‐PEG antibodies and adverse immune reactions [39, 40]. Furthermore, PEG lacks cell adhesion sites and intrinsic biological activity, necessitating the introduction of other groups to promote cell adhesion or enhance biological function.

### 2.3 Composite Materials

Composite hydrogels are multifunctional systems formed by combining two or more natural polymers, synthetic polymers, or inorganic nanomaterials through physical blending, chemical crosslinking, or in situ composite methods. Their primary advantage lies in integrating the characteristics of different components to compensate for the deficiencies of single materials, achieving synergistic optimization in mechanical properties, biocompatibility, and stimulus responsiveness. This makes them indispensable for preparing HMs. For example, a bioink composed of GelMA HMs and CS microspheres effectively mimics neural network systems in both microscale and macroscale environments [41].

The incorporation of inorganic nanomaterials represents a key strategy for functionalizing composite HMs. Composite microspheres based on hydroxyapatite nanoparticles and alginate have demonstrated outstanding performance in bone tissue engineering. Hydroxyapatite not only enhances the mechanical properties of microspheres but also promotes osteogenic differentiation [42]. Similarly, manganese dioxide (MnO_2_) composite nanoparticles incorporated into HAMA microspheres promote the regeneration and repair of degenerated intervertebral discs in ischemic tissues [43]. Multifunctional composite microspheres can also achieve intelligent regulation through stimulus‐responsive components. For instance, pH‐responsive alginate/calcium carbonate composite hydrogel microparticles have been designed for the sustained dual release of antibiotics (rifamycin) and growth factors (basic fibroblast growth factor; bFGF), thereby promoting wound healing [44]. Such composite systems, through synergistic interactions between their components, overcome the limitations of single materials in mechanical properties, bioactivity, or functional regulation and broaden the application scope of HMs in drug delivery and tissue repair.

### 2.4 Influence of Material Selection on HM Properties

HMs are primarily composed of natural polymers (e.g., alginate, gelatin, HA) or synthetic polymers. The choice of material directly determines the core properties of the microspheres. Degradability, a key property of HMs, influences tissue regeneration by regulating drug release rates. This is particularly important given the differing regeneration cycles of tissues: bone tissue healing requires weeks to months, whereas cartilage tissue regeneration may take years [50, 51].

The mechanical properties of HMs are another crucial factor. They must match the mechanical characteristics of the target tissue to avoid adverse reactions and promote regeneration. For example, bone tissue, because of its mineralized collagen, requires microspheres with high stiffness for structural support [52]. Cartilage tissue requires microspheres with a lower elastic modulus to preserve proper pressure distribution in the joint cavity [53]. Soft tissues such as nerves and skin require microspheres with high elasticity to match surrounding tissue mechanics and prevent complications such as compartment syndrome [54]. GelMA‐based HMs can be tuned to an elastic modulus between 2.43 and 68.2 kPa by adjusting ultraviolet crosslinking time and gel concentration, thereby mimicking the physiological stiffness of bone marrow, blood vessels, and immature bone [55].

Pore size also significantly influences HM performance, as it affects cell infiltration, nutrient transport, and tissue regeneration efficiency. It further regulates the differentiation potential of MSCs. For example, alginate‐based HMs with smaller pore sizes (80–120 µm) effectively promote MSC chondrogenic differentiation [56]. HMs with intermediate pore sizes (∼200 µm) optimize the in vitro angiogenic differentiation of endothelial progenitor cells, while larger pore sizes (∼300 µm) favor vascular formation in vivo [57]. Additionally, porous structures enhance platelet aggregation and cell adhesion, thereby improving hemostatic efficiency [58].

Crosslinking methods and parameters also impact HM biocompatibility. For HMs based on gelatin, HA, and PEG, factors such as light intensity, photoinitiator type, and photoinitiator concentration influence the viability of encapsulated cells during ultraviolet‐induced crosslinking [59]. Moreover, using patterned masks to block ultraviolet light or adjusting light intensity allows precise crosslinking of microspheres in specific regions, enabling control over microsphere size and shape and the construction of complex microstructures [30].

## 3 Preparation of HMs

**FIGURE 2:**
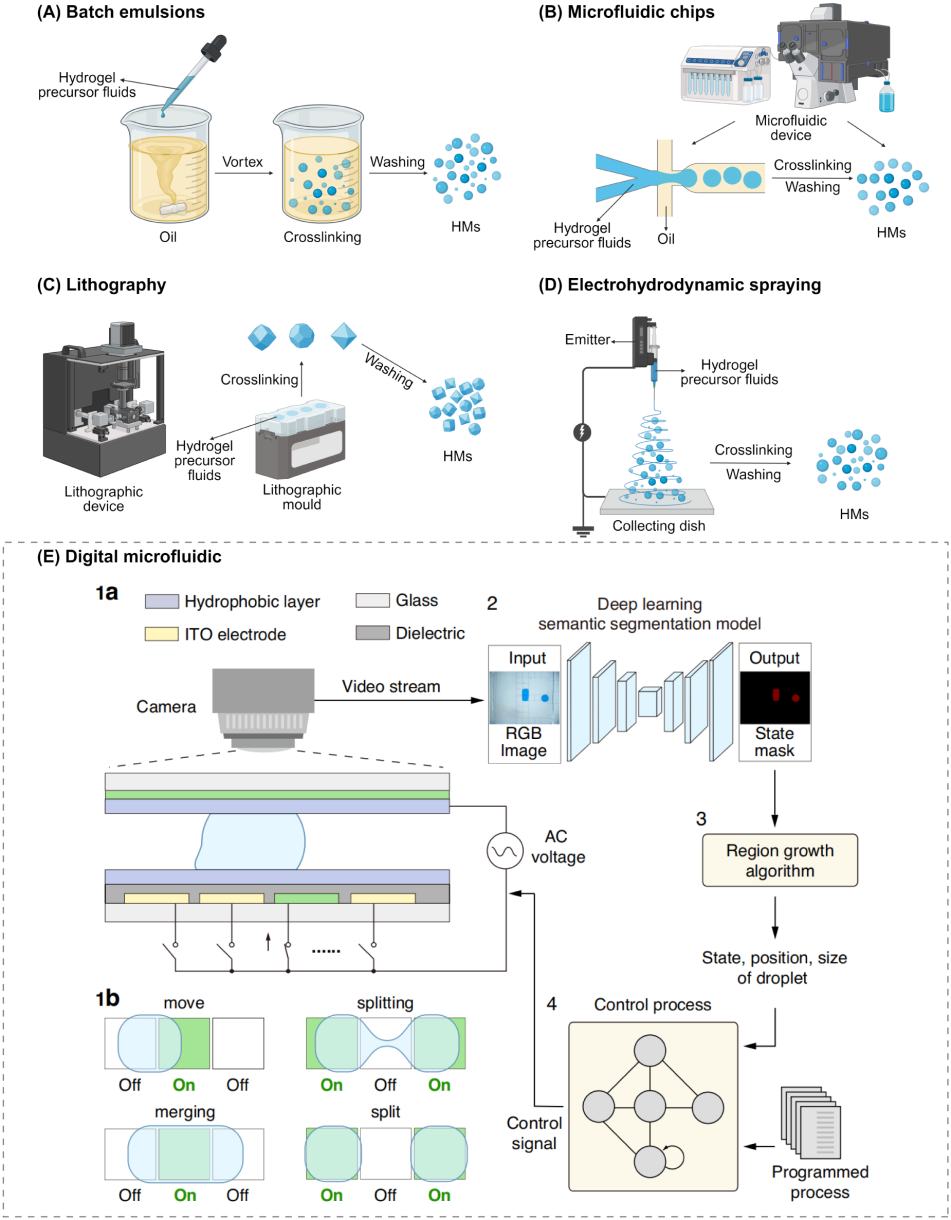
Fabrication of HMs. Techniques include: (A) Batch emulsions, mixing immiscible liquids to form cross‐linkable droplets. (B) Microfluidic chips, using microfluidic devices for production. (C) Lithography, fabricating microscale hydrogels via molds. (D) Electrohydrodynamic spraying, generating charged droplets via electrical forces for subsequently HMs formation. (E) Artificial intelligence‐assisted digital microfluidics system. Reproduced with permission [60]. Copyright 2024, The authors.

Based on the type of hydrogel and crosslinking method, various techniques have been developed for the preparation of HMs, including batch emulsion, photolithography, microfluidic chip, and EHD spraying. Each strategy offers distinct advantages in terms of operational simplicity, microsphere formation speed, and control over microsphere complexity. For example, batch emulsion technology is favored for its simplicity, low equipment requirements, and relatively fast production speed. When higher precision in HMs size and structure is required, more advanced strategies such as microfluidic chip technology and photolithography are preferred. Selecting the appropriate method depends on application requirements and material characteristics. With ongoing technological advances and deeper research, HM fabrication methods continue to evolve to meet the increasingly complex demands of biomedical and bioengineering applications. Therefore, we summarize several commonly used fabrication techniques for preparing HMs (Figure 2) and describe their respective parameter characteristics in Table 2. This provides researchers with a reference for selecting suitable preparation strategies for future studies, potentially enhancing efficiency and driving innovation in the field.

**TABLE 2:** Comparative analysis of parameters for HMs fabrication techniques.

Production method	Production rate	Size range	HMs size coefficient of variation (CV%)	Automation and costs	Cells compatibility
Batch emulsion	High	2.5–100 µm [61] [62],	Large variation [61, 63]	Fully manual operation with low cost	60–80% viability [63]
Microfluidic chip	Average	10–50 µm [64] [65],	Low variation (1.3–3.9%) [64, 65, 66]	Highly automated with moderate cost	>80% viability [67, 68]
Lithography	Low	<1 µm possible [69]	Low variation (1–3.3%) [69, 70]	Moderately automated with moderate cost	68–92% viability [71, 72]
Electrohydrodynamic spray	Average	3–300 µm [73, 74]	Large variation 40–60% [73, 75]	Highly automated with moderate cost	60–80% viability [75, 76]
Digital microfluidic	Low	56–100 µm [77, 78, 79]	Low variation (2.7–5.6%) [60, 79]	Highly automated with high cost	>90% viability [77, 78, 79]

### 3.1 Batch Emulsion Technology

Batch emulsion technology is one of the most commonly used methods for preparing HMs. It includes two main types: emulsion polymerization and phase separation of biopolymers. The principle involves mixing immiscible oil and hydrogel precursor solutions to form droplets, which are homogenized by mechanical agitation. These droplets are subsequently crosslinked through methods such as photopolymerization or temperature changes, resulting in HMs [80, 81].

The main advantages of this method include simplicity, low equipment requirements, and high yield, making it suitable for synthesizing HMs. Emulsion polymerization is also compatible with biological agents, enabling the uniform dispersion of cells or bioactive substances in the hydrogel precursor before emulsification, which enhances application potential. For example, Franco et al. [63] successfully prepared HMs loaded with highly active cells, maintaining 60–80% viability.

However, HMs produced by emulsion polymerization typically exhibit larger sizes and broader distributions due to uneven energy input during mixing [82]. This polydispersity can cause significant variations in performance between batches [83], affecting their consistency in applications such as drug release or biological carriers. Optimizing mixing conditions and controlling droplet size are therefore essential for improving product quality. Moreover, HMs produced by emulsion polymerization require extensive washing to remove residual oil, which may compromise performance and bioactivity.

To avoid oil‐phase interference, researchers have developed all‐aqueous two‐phase separation methods. These rely on interactions between biopolymers, with crosslinking in the internal aqueous phase triggered by environmental changes, resulting in microgel beads. Phase separation can occur via electrostatic complexation or thermodynamic incompatibility. Electrostatic complexation exploits interactions between oppositely charged biopolymers to form aggregates [84, 85, 86, 87]. Mechanical force then generates a “water‐in‐water” emulsion, and gelation is induced by adjusting solution conditions. This method is well suited for encapsulating bioactive substances, though stability is highly sensitive to pH and ionic strength, making dissociation likely [88, 89]. Thermodynamic incompatibility, in contrast, arises from repulsion between biopolymers, producing phases of differing concentrations [84, 87, 90, 91]. Under shear force, these phases form “water‐in‐water” emulsions. However, dispersed particles often coalesce, which can be mitigated by stabilization strategies such as crosslinking or biopolymer coating [92, 93].

### 3.2 Microfluidic Chip Technology

Microfluidic chip technology offers significant advantages for HM fabrication, particularly in its precise control of fluid flow and maintenance of stable laminar conditions [94, 95]. This technique exploits the interaction between oil and aqueous phases, producing uniform micrometer‐scale droplets by adjusting flow rates and microchannel geometries [65, 96]. Microfluidics can generate monodisperse droplets with a dispersity index as low as 1.3–3.9% [64, 65, 66, 97], providing excellent conditions for HM production.

**FIGURE 3:**
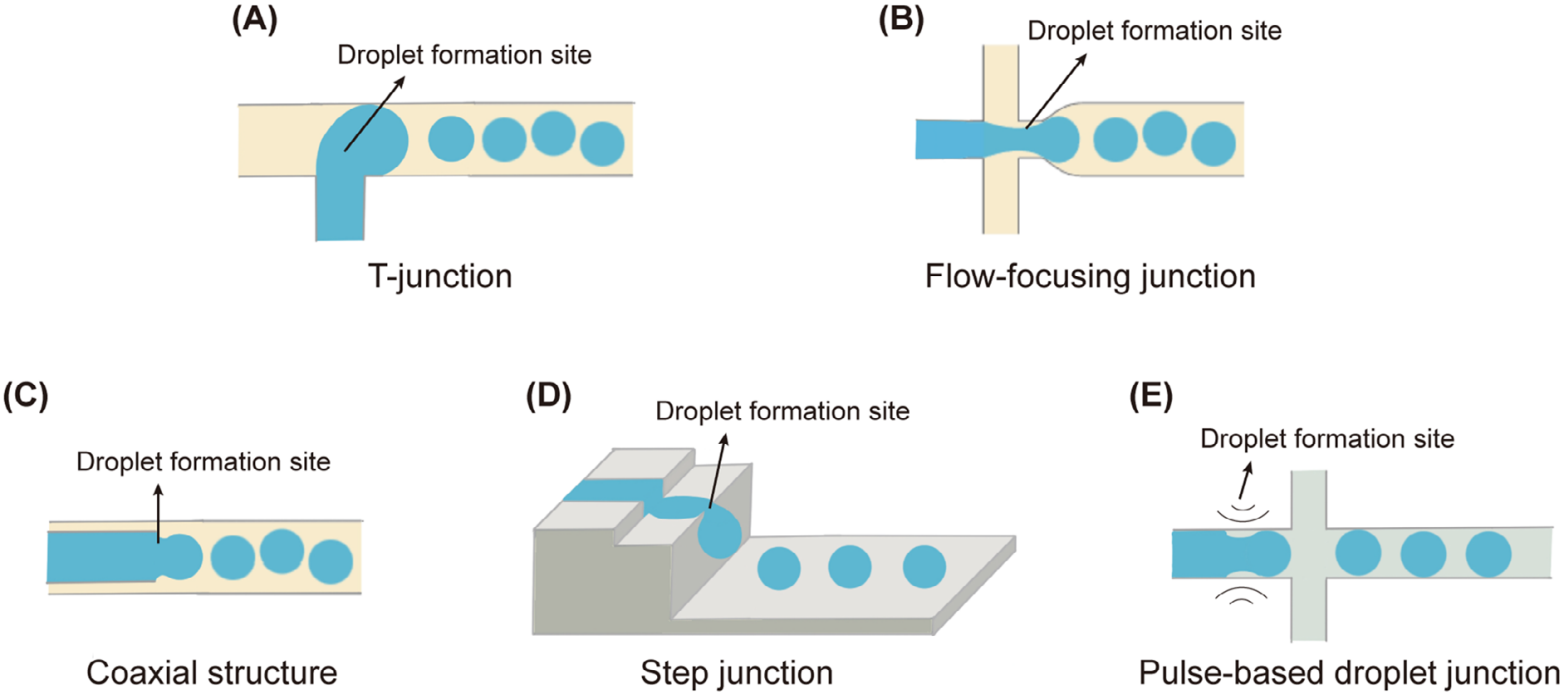
Schematic representation of droplet formation in microfluidic chips featuring various geometries of junctions. (A) T‐junction, (B) flow‐focusing junction, (C) coaxial structure, (D) step junction, and (E) pulse‐based droplet junction.

Microfluidic chips are fabricated from materials such as PDMS, silicon wafers, glass, and polymers. PDMS is particularly common due to its ease of processing, optical transparency, and elasticity. Chip design allows for complex microchannel architectures, such as T‐junctions, flow‐focusing junctions, or coaxial geometries, that enhance droplet formation speed and precision. Different junction geometries yield diverse droplet formation patterns [18, 98, 99, 100]. Common junction designs are outlined in Figure 3. The T‐junction features 90° intersection of continuous and dispersed phase channels, where the continuous phase shears the dispersed phase into droplets [101, 102, 103]. The flow‐focusing junction, formed by the intersection of continuous and dispersed phase channels, periodically compresses and shears the dispersed phase for efficient droplet generation [104, 105, 106]. the coaxial structure consists of two coaxial channels (outer channel as continuous phase, inner channel as dispersed phase), with droplets forming via Rayleigh–Plateau instability [105, 107]. The step junction has a step‐like structure, where droplets form due to Laplace pressure imbalance across the step [108, 109]. The pulse‐based droplet junction uses periodic mechanical pulses to generate droplets in pure aqueous systems [110, 111, 112], its oil‐free design preventing damage to bioactive substances (such as cells and proteins) and thus suitable for high‐biocompatibility scenarios like cell encapsulation.

Moreover, precise microchannel designs enable HMs with core–shell or multichamber structures, enhancing functionality and application potential. Studies show that microfluidic‐prepared HMs perform well in drug delivery, as they can encapsulate therapeutic agents and release them in a controlled manner.

Despite the advantages of microfluidic technology, it also faces several challenges, including low production volumes and difficulties in large‐scale manufacturing [113, 114, 115]. These limitations primarily result from the structural constraints of microfluidic devices and the viscosity requirements of hydrogel precursor solutions. To address these issues, air microfluidics has been developed as an alternative to chip‐based systems. This technique involves two microscale liquid streams being injected together and colliding to form droplets [116, 117]. Compared with emulsion‐based microfluidic chips, air microfluidics achieves higher liquid flow rates, producing particles at rates 10–100 times faster [116, 117]. Furthermore, microchannels in conventional chips are prone to clogging, which reduces equipment lifespan and compromises production stability [1, 115]. Future research should focus on optimizing device design and developing new materials to overcome these bottlenecks, thereby enhancing the application potential of microfluidic technology in HMs preparation. Improved device engineering and process optimization are expected to increase production efficiency and expand application scope. While microfluidic chips rely on surface tension to form spherical droplets, the fabrication of nonspherical particles requires alternative techniques such as photolithography, discussed below.

### 3.3 Lithography Technology

Lithography technology shows considerable potential for HM fabrication, with three main approaches: photolithography, imprint lithography, and flow lithography [118]. These techniques enable precise control of microsphere diameter and geometry by photopolymerizing hydrogels on microscale templates, producing highly uniform and monodisperse HMs [71, 119, 120]. Nichol et al. [71] demonstrated that hydrogels fabricated via photolithography not only maintain consistent size and shape but also promote cell adhesion, migration, and proliferation, highlighting their broad potential in tissue engineering.

Although photolithography provides strict control of geometry, it faces challenges such as slow fabrication speed and limited penetration depth [1]. In imprint lithography, an additional challenge is the removal of crosslinked microspheres from molds, which restricts the complexity of achievable features. To overcome these issues, multiphoton light sources and advanced lithography components have been developed, enabling the creation of complex three‐dimensional structures. Commonly used materials include acrylate‐ and methacrylate‐based hydrogels, which are biocompatible and can be rapidly crosslinked with photoinitiators. While photolithography generally yields lower production volumes, microfluidic photolithography offers higher throughput by integrating lithography with microfluidic technology. Continuous optimization of polymerization conditions and material properties will further strengthen photolithography as a promising method for controlled HM fabrication and biomedical applications.

### 3.4 EHD Spray Technology

EHD spraying, originally adapted from the electronics manufacturing industry, has recently emerged for preparing highly uniform, monodisperse HMs. Standard equipment includes a syringe pump, high‐voltage power supply, conductive nozzle, and collection device [76, 121]. During operation, the hydrogel precursor solution forms a Taylor cone, that is, the conical tip formed when surface tension and electric field forces balance, at the nozzle. Droplets are then ejected into the collection device. By adjusting parameters such as voltage, nozzle size, and flow rate, droplet size can be precisely controlled, down to 1–2 µm [73]. In addition, EHD spraying is compatible with cell encapsulation, as droplets can rapidly crosslink with encapsulating agents to form HMs. Gansau et al. [75] successfully used this method to prepare cell‐loaded HMs with high viability.

Despite these advantages, EHD spraying has drawbacks, including the need for specialized equipment, reliance on high‐voltage power supplies, and challenges in droplet collection. This method, which generates charged droplets through a high‐voltage electrostatic field [122, 123], offers advantages such as high purity and operational simplicity compared with traditional techniques and is applicable to a wide range of polymers. However, electrospray microspheres still face challenges, including uncontrolled morphology, rapid drug release from polymer‐based microspheres, and complex preparation processes for composites.

### 3.5 Emerging Technologies

Continuous innovation in microfluidics has driven major breakthroughs in HMs preparation. By integrating high‐precision manipulation, intelligent recognition, and targeted screening, preparation efficiency and application potential have been greatly improved.

Droplet‐based digital control is exemplified by the integrated droplet‐digital microfluidic platform [77]. This system combines high‐throughput droplet generation with on‐demand digital control. It first generates droplets containing single cells or microbeads, sorts them by fluorescence intensity, and then merges target droplets with recovery buffer to achieve precise single‐cell recovery. The platform achieves high recovery rates, 93.5% for multibead recovery, 87% for single‐bead recovery, and >80% even for rare cells (1:400 or 1:1000), substantially outperforming traditional approaches. This highlights its unique value for single‐cell analysis, antibody discovery, and rare‐cell research.

The AI‐assisted digital microfluidic system μDropAI developed by Guo et al. [60] exemplifies further innovation. Using a deep‐learning semantic segmentation algorithm, μDropAI accurately identifies multistate droplets despite variations in illumination, color, or shape. Its integration of closed‐loop feedback enables real‐time regulation of droplet operations, allowing automatic correction of failed actions and improved volume consistency of split droplets. This provides a versatile solution for automated, precise digital microfluidic platforms.

Additionally, machine vision systems based on the Deformable DETR algorithm have advanced intelligent droplet analysis [124]. This technology achieves <4% relative detection error and >94% accuracy across multiscale and multiscenario applications. The associated web‐based tool MDIA supports transfer learning, enabling droplet characterization by parameters such as diameter and frequency. With ongoing data accumulation, its universality continues to expand, providing robust database support for droplet microfluidics.

Similarly innovative is the platform developed by Tang et al. [78], which integrates a double‐helix focusing module and a flow‐resistance enrichment module. This platform arranges cells into a single streamline through an eight‐loop double‐helix channel, and, together with a serpentine unit that removes 50% of the excess aqueous phase, achieves single‐cell encapsulation efficiencies of 79.2% for 15 µm microspheres and 72.2% for MDA‐MB‐231 cells. These results surpass the theoretical limit of passive encapsulation based on the Poisson distribution (approximately 37%), providing an efficient strategy for preparing HMs with uniform cell loading. Nakamura et al. [79] reported an advance with their integrated optical sorting and piezoelectric‐driven system. This system aligns cells in single file using sheath flow, identifies target cells through multiparameter optical detection (forward/backward scatter and fluorescence), and uses piezoelectric elements to trigger pressure pulses that encapsulate target cells into water‐in‐oil droplets, while nontarget cells are directed to a waste channel. Wei et al. [125] developed an integrated digital droplet PCR platform that combines droplet generation, PCR amplification, and droplet detection on a single chip. This system automatically analyzes data after sample loading, fulfilling the clinical requirement of “sample in, result out.”

The integration of digital control, intelligent recognition, and targeted screening has enhanced the fabrication efficiency and uniformity of HMs while broadening their applications in personalized drug delivery, high‐throughput cell detection, and rare cell analysis. These advances establish a foundation for the large‐scale implementation of microfluidic technology in the biomedical field.

### 3.6 Technical Bottlenecks

Although various HMs fabrication techniques each exhibit unique advantages, their technical bottlenecks still restrict broader application. Batch emulsion technology is limited by wide size distributions and batch‐to‐batch variations caused by mechanical mixing [82]. In all‐aqueous systems, the electrostatic complexation method is highly sensitive to pH and ionic strength, making it prone to dissociation [88, 89], while systems based on thermodynamic incompatibility face coalescence of dispersed phase particles [91], which requires stabilization through crosslinking or coating [92, 93]. Microfluidic chip technology allows high‐precision control (with dispersity indices as low as 1–2%) [66, 97], but it is constrained by low production yields, susceptibility to microchannel clogging, and uniformity limited to spherical structures, making large‐scale manufacturing difficult [101, 102, 103]. Lithography excels in geometric control but suffers from low fabrication efficiency, and the difficulty of mold detachment in imprint lithography restricts structural complexity. Even when combined with microfluidics, its limitations persist [1]. EHD spraying requires specialized equipment (e.g., high‐voltage power supplies) and faces challenges including difficult droplet collection, poor control of microsphere morphology, and complex processes for composite microspheres [122, 123]. Collectively, these bottlenecks manifest as limitations in size uniformity, scalability, operational complexity, and functional adaptability. They are driving the optimization of existing techniques and exploration of new strategies to meet the demand for high‐precision, multifunctional HMs in biomedical applications.

## 4 Applications of HMs

**FIGURE 4:**
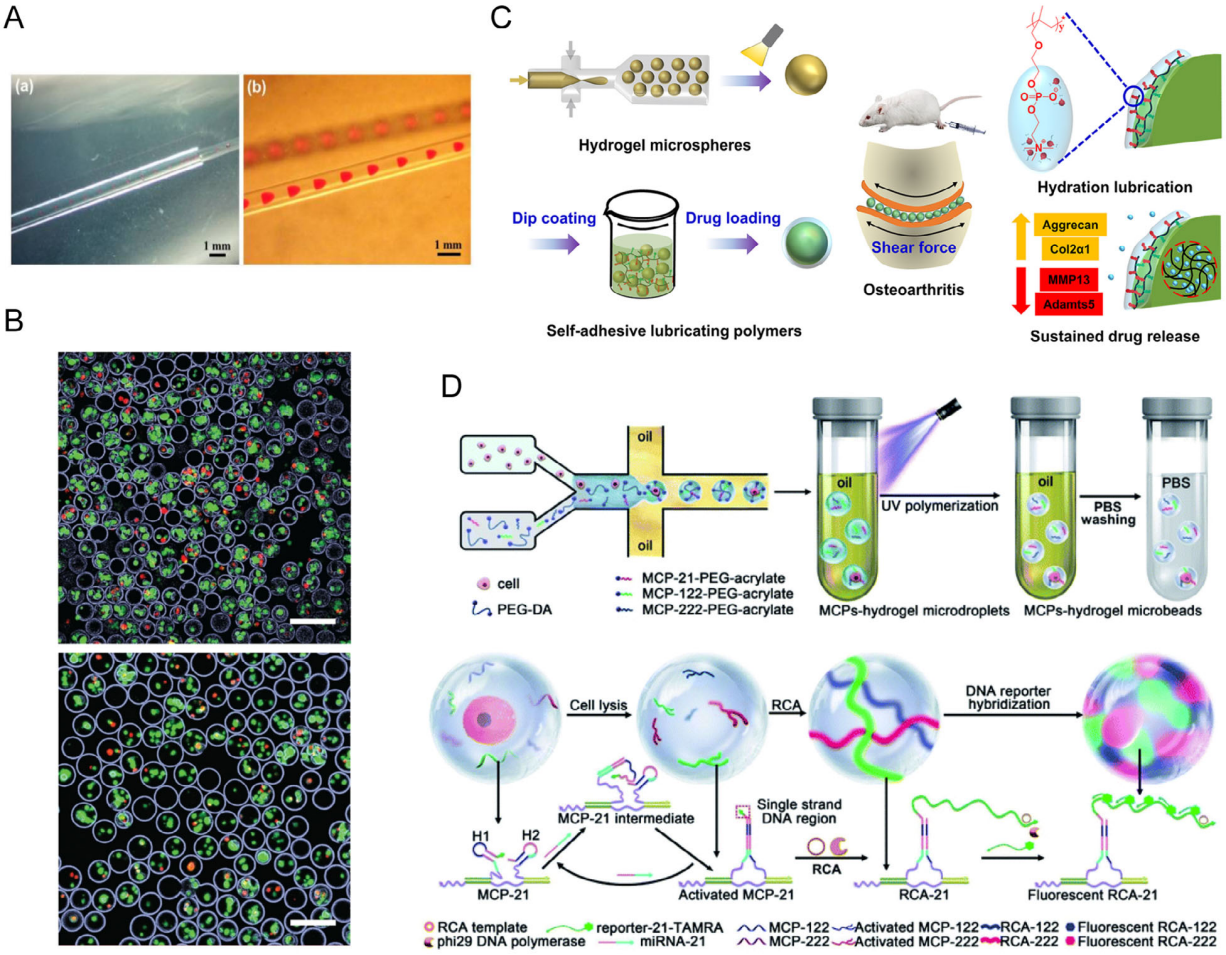
Representative images of HMs in application. (A) The droplet in the capillaries. Reproduced with permission [126]. Copyright 2023, The authors. (B) Representative images are shown for cells microencapsulated on both 35 µm (up) and 100 µm (down) nozzle widths (scale bar 200 µm). Reproduced with permission [67]. Copyright 2018, The authors. (C) Biomimetic injectable HMs with enhanced lubrication and controllable drug release for the treatment of osteoarthritis. Reproduced with permission [127]. Copyright 2021, The authors. (D) Single cell multi‐miRNAs quantification with hydrogel microbeads for liver cancer cell subtypes discrimination. Reproduced with permission [128]. Copyright 2022, The authors.

The unique properties of HMs hold promise for diverse biomedical applications. They enable minimally invasive delivery of biomaterials via injection, allowing controlled release of bioactive substances at injury sites. This delivery method enhances the clinical utility and efficacy of HMs, facilitating their widespread adoption. This section discusses applications including drug delivery, cell and gene therapy, hydrogel–nanoparticle integration for synergistic therapy, lubrication modification of HMs, and diagnostic and imaging applications. A representative image is shown in Figure 4.

### 4.1 Drug Delivery Systems

#### 4.1.1 Diffusion‐Control Mechanisms of HMs

Many drugs used for tissue repair and regeneration face challenges during delivery. Hydrogels show strong potential in this area because they protect, transport, and locally release bioactive factors and drugs in a controlled manner [129]. Hydrogel‐based delivery not only overcomes limitations of traditional methods (e.g., high doses and repeated administrations required for oral or intravenous delivery) but also allows minimally invasive injection, avoiding the trauma of scaffold implantation. However, early burst release of growth factors may occur, potentially causing toxic effects in surrounding tissues [130, 131].

Factors such as HMs particle size and micropore diameter influence sustained release of growth factors. Particle size is a key determinant: larger microspheres exhibit more sustained release due to greater diffusion distances compared with smaller ones [83, 132]. Similarly, pore size affects release kinetics, smaller micropores promote gradual release [133, 134], which can be achieved by increasing polymer concentration and crosslinking density. At the molecular level, covalent or noncovalent interactions between growth factors and hydrogels can also enhance drug loading and slow release.

A series of interactions that contribute to stabilizing and sustaining drug release may occur between growth factors, drugs, and HMs, including covalent bonding, electrostatic adsorption, and hydrophobic interactions [135, 136]. Heparin, a highly sulfated polysaccharide, can reversibly bind proteins and has been used for the sustained delivery of various growth factors [137]. Similarly, sulfation of common hydrogels containing furan acids (such as HA and alginate) enhances growth factor binding [138, 139, 140]. For instance, stronger binding has been achieved in alginate hydrogels [141, 142]. To summarize the reasons for this strong binding: sulfation introduces negative charges that create strong electrostatic attractions with positively charged growth factors. In addition, sulfate groups can form hydrogen bonds with amino or hydroxyl groups in growth factors, further enhancing binding stability. Moreover, sulfation also increases hydrophilicity and may lead to structural changes in the hydrogel, expanding the surface area or spatial configuration available for binding growth factors. Fibronectin (Fn) contains binding sites composed of segments 6FnI–1FnII–2FnII–7FnI–8FnI–9FnI, which have been utilized for strong anchoring to HMs [143]. Fn can also efficiently bind to various growth factors through its heparin‐binding sites formed by the 12FnIII–13FnIII–14FnIII segments, enhancing the adsorption of proteins [144] and cells [145].

The unique structures and functions of different organs make it challenging for drugs to exert sustained and effective actions. For instance, synovial tissue within joint cavities secretes synovial fluid, which accelerates drug clearance. In the respiratory tract, specific particle sizes are required to prevent premature uptake and clearance by macrophages. Oral medications for intestinal diseases must also withstand degradation by stomach acid. HMs have emerged as an ideal therapeutic tool in regenerative medicine. Kartogenin (KGN), a small‐molecule drug used in musculoskeletal disorders (MSDs) [146], demonstrates significant therapeutic efficacy but faces challenges such as uneven distribution and poor solubility due to its hydrophobic nature. Modifying KGN with HMs offers a promising strategy to overcome these limitations. Anti‐inflammatory and antioxidant small‐molecule drugs, such as celecoxib and diclofenac, have also shown strong therapeutic effects. Additionally, traditional Chinese medicine compounds with anti‐inflammatory and antioxidant properties, including liquiritin, vanillin, dihydromyricetin, and hydroxychloroquine, are emerging as potential research hotspots for future studies.

#### 4.1.2 Environment‐Responsive Release of HMs

By engineering environment‐responsive HMs to release drugs under specific physiological pH conditions or inflammatory environments, the timing and location of drug or bioactive substance release can be more precisely controlled. Alginate hydrogel fibers encapsulating pH‐sensitive polymer microspheres were fabricated using a coaxial capillary microfluidic device [126] (Figure 4A). After modification with carboxymethyl cellulose, the alginate hydrogels exhibited enhanced pH sensitivity, which regulated the diffusion rate of molecules released from the inner microspheres. These microsphere fibers show potential for selective drug release in acidic environments such as tumor or inflammatory sites, making them promising intelligent surgical dressings with protective effects on normal tissues. Ionotropic gelation was used to fabricate tragacanth gum/β‐cyclodextrin/SA HMs [147], which efficiently encapsulate hydrophobic drugs. These microspheres exhibit distinct swelling behaviors and drug release profiles under varying pH conditions, enabling targeted drug delivery in specific gastrointestinal environments. Moreover, they can regulate drug release rates, thereby reducing adverse side effects and improving therapeutic efficacy. HMs incorporating synthetic polyphenolic antioxidants undergo accelerated degradation in the acidic microenvironment of degenerated intervertebral discs, releasing active components to enhance antioxidant efficacy [148].

A microenvironment‐responsive bilayer HM was fabricated using microfluidic technology. The outer shell, composed of GelMA, rapidly responds to the OA microenvironment to release celecoxib, while the inner core of chondroitin sulfate methacrylate gradually releases chondroitin. This synergistic system achieves both anti‐inflammatory effects and cartilage repair, providing a novel strategy for OA treatment [149]. A hypoxia‐ and MMP‐13 dual‐responsive HM has also been shown to significantly alleviate OA progression by targeting the inflammatory microenvironment [150]. By combining microfluidic methods with photopolymerization, cartilage‐targeting peptides and ROS‐responsive nanoparticles were integrated into the hydrogel matrix to develop cartilage‐targeting HMs with ROS‐responsive capabilities [151]. These microspheres can specifically target and repair cartilage in OA models.

Porous HMs with responsive functionalities can intelligently release loaded drugs in response to external stimuli such as temperature, magnetic fields, electric fields, and light. Thermo‐responsive agarose HMs encapsulating methotrexate‐packaged TMPs and black phosphorus quantum dots (BPQDs) have been fabricated via microfluidic technology, enabling synergistic therapy for malignant ascites under near‐infrared irradiation [152]. HMs incorporating barium titanate piezoelectric particles can generate ROS under ultrasound induction. By activating the cGAS–STING pathway, they achieve dual anti‐infective efficacy through physical debridement and immune activation [153].

### 4.2 Cell and Gene Therapy

Transplantation of seed cells to repair damaged tissues is a primary strategy in regenerative medicine. However, the limited number of seed cells that can be delivered, combined with their poor survival after transplantation, hinders progress in this field. Hydrogels serve as scaffolds by providing adhesion sites for seed cells, and modifying or regulating hydrogel properties can significantly enhance cell survival. Conventional bulk hydrogels may restrict the transport of oxygen, nutrients, and metabolic waste due to their larger size [153]. In contrast, HMs provide higher surface‐to‐volume ratios, which facilitate improved transport. Moreover, the noninjectability of certain hydrogels limits their suitability for minimally invasive treatments. These limitations of bulk hydrogels have generated strong interest in using HM systems for cell delivery. In this context, we discuss the advancements in HMs for cell delivery.

Using HMs for cell delivery facilitates the transport of oxygen and nutrients, and they can be implanted into damaged areas through minimally invasive injection methods. While embedding cells in HMs, it is essential to consider strategies for enhancing the survival rate of the encapsulated cells. The gelation method of the microspheres, the presence of free radicals, chemical substances, and potential physical shear forces can all affect cell viability. Traditional oil‐in‐water emulsion techniques for encapsulating cells may reduce cell survival due to the presence of potentially harmful oils and surfactants. Additionally, the subsequent centrifugation and washing steps to separate the HMs from the oil can also impact cell viability and decrease the yield of the microsphere particles. Thus, researchers developed a series of filtration methods to quickly separate the cross‐linked HMs from the oil phase, significantly enhancing cell survival rates [154, 155]. A double microfluidic water‐oil‐water method was employed to prepare cell‐loaded GelMA HMs with ultra‐thin oil shells. These oil shells spontaneously dehydrated when transferred to an aqueous solution, thereby reducing the time cells were exposed to the oil phase and improving cell survival rates [155]. An oil‐free, all‐aqueous microfluidic method also generated HMs based on phase separation characteristics. Researchers developed an oil‐free microfluidic device capable of producing alginate‐based HMs loaded with cells, achieving a survival rate of up to 91% [156, 157]. The concentration of HM materials, such as alginate, can influence cell viability. Higher concentrations of alginate‐based HMs were found to significantly enhance the post‐thaw recovery and viability of cryopreserved cells [158].

For the microfluidic chip fabrication method, the width of the chip channels required careful optimization, as narrower channels (35 µm, with a survival rate of 71%) resulted in significantly lower cell survival rates compared with wider channels (100 µm, with a survival rate of 90%) [67], as shown in Figure 4B. The distribution of seed cells within HMs is another important consideration in tissue engineering therapies. In the preparation of HMs using emulsion and photolithography techniques, increasing the cross‐linking speed of the hydrogel prevented cells from settling in the gel precursor solution [1, 159, 160]. Voltage‐driven spray technology also presents challenges in achieving uniform cell distribution; excessively high voltages can cause cells to aggregate at the edges of the HMs, which negatively affects cell viability [74, 75].

Gene therapy, particularly gene editing via the CRISPR/Cas9 system, offers a solution to genetically modify target cells at the genomic level, thereby overcoming the limitations of traditional therapeutic approaches. Using CRISPR/Cas9 technology, the research team developed a cell‐affinity peptide (CAP)/FGF18–hyEXO hybrid vector combined with HAMA HMs [161]. This system enables efficient delivery and gene editing of chondrocytes, promoting chondrocyte proliferation, ECM synthesis, and cartilage matrix regeneration. Delivery of TPCA‐1 via GelMA–silk fibroin HMs selectively inhibits the IKKβ/NF‐κB signaling pathway. This not only suppresses the formation of ectopic cartilage/bone but also helps to mitigate the inflammatory microenvironment, thereby promoting tendon repair [162].

### 4.3 Multifunctional Composite Systems

#### 4.3.1 The Integration of HMs and Nanoparticles for Synergistic Therapy

**FIGURE 5:**
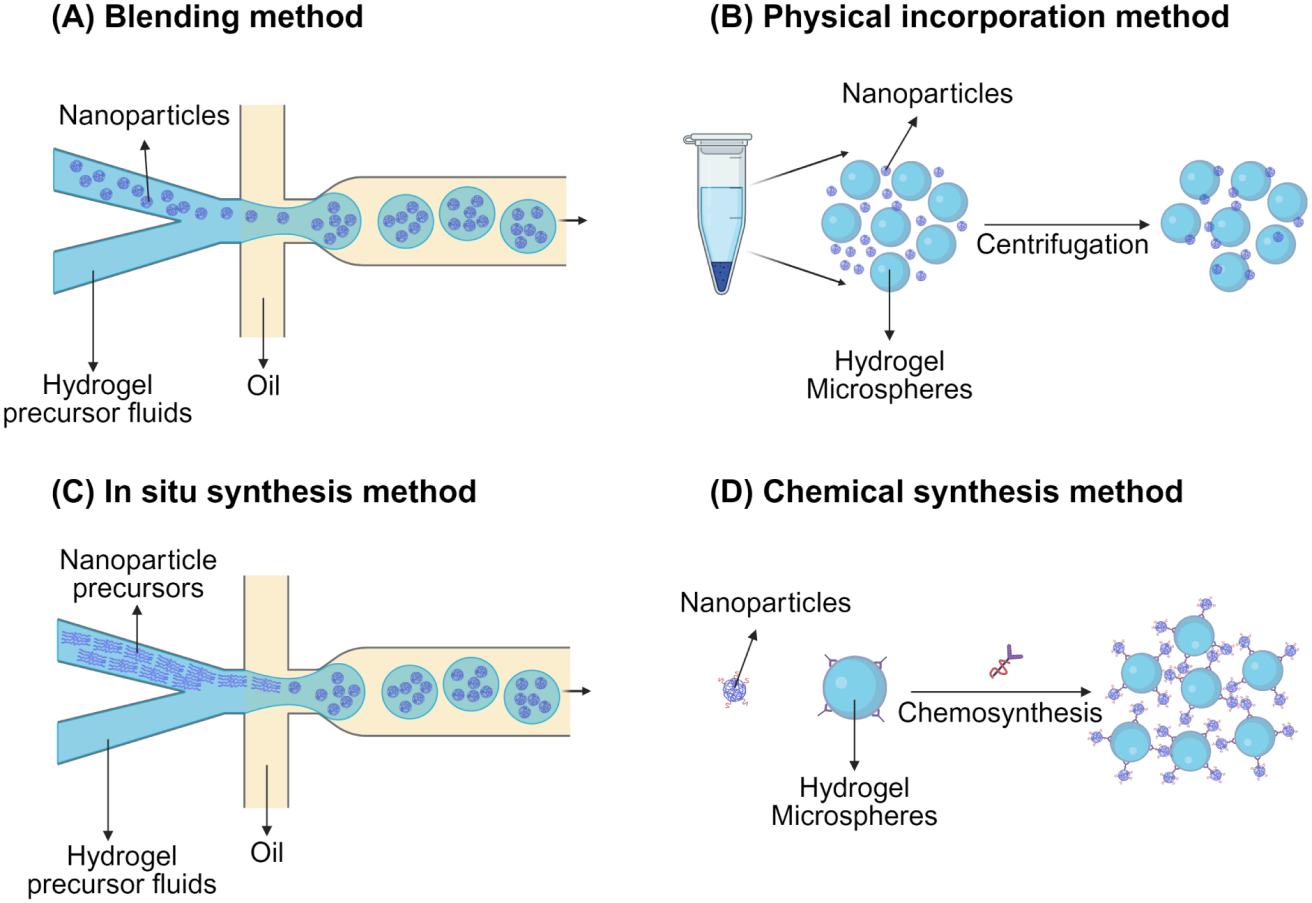
Four strategies for incorporating multifunctional nanoparticles into HMs. Including the blending method, physical incorporation method, in situ synthesis method, and chemical synthesis method.

Nanoparticles have a wide range of applications, including improving the absorption rates of poorly soluble drugs and enabling targeted drug release. However, due to their small particle size, they can easily penetrate cell membranes and disrupt cellular functions. Additionally, nanoparticles are susceptible to external factors during preparation and storage, which can affect their stability and efficacy. The incorporation of nanoparticles allows composite HMs to leverage the advantages of both components. For instance, HMs can reduce the toxic side effects of nanoparticles and control their release kinetics, while also enhancing the biocompatibility of the nanoparticles. Conversely, nanoparticles can enhance the stability of hydrogels, improve their mechanical properties, and even provide targeted functionalities through specific strategies, such as the introduction of chemical bonds. At present, there are four common strategies for incorporating multifunctional nanoparticles into HMs (Figure 5). The blending method involves mixing nanoparticles with a hydrogel precursor solution, followed by gelation to form nanoparticle‐reinforced HMs. The physical incorporation method entails directly integrating nanoparticles into preformed HMs. In situ synthesis involves mixing nanoparticle precursors into the hydrogel or hydrogel precursor solution, leading to the in situ generation of nanoparticles. Last, the chemical synthesis method uses chemical reactions to create bonds between the HMs and the nanoparticles, facilitating their incorporation.

The integration of HMs and nanoparticles shows significant promise in the fields of regenerative medicine and oncological disease management. Photothermal therapy utilizes photothermal conversion agents (such as black phosphorus and metal nanoparticles) to absorb near‐infrared light and convert it into thermal energy, enabling the elimination of bacteria or ablation of diseased cells. As carriers, HMs can deliver these photothermal conversion agents in a targeted manner while controlling drug release, thereby achieving a synergistic photothermal–chemical therapeutic effect. Hydrogels loaded with copper‐containing polydopamine (PDA) nanoparticles can kill bacteria through photothermal effects, while releasing Cu^2^⁺ to inhibit infections over the long term and promote the healing of diabetic wounds [163]. In a self‐assembled HM scaffold based on charge interactions, black phosphorus is loaded into CS methacryloyl microspheres, while growth factors are loaded into HA microspheres. Under near‐infrared light irradiation, the thermal energy generated by black phosphorus accelerates drug release, enabling photothermal–chemical synergistic wound repair [164]. HMs loaded with BPQDs can achieve on‐demand control of antitumor drugs through mediating photothermal stimulation, regulate the tumor immune microenvironment, and thereby realize synergistic chemotherapy, photothermal therapy, and immunotherapy for malignant ascites [152].

#### 4.3.2 HMs with Lubricating Functionality in Joint Diseases

Many diseases require tissue‐engineered scaffolds to have lubricating functionality, especially in the case of musculoskeletal injuries. The unique micron‐sized spherical structure of HMs can be used to alter the frictional interactions in moving organs, such as articular cartilage and tendons. Inspired by mechanical ball bearings, HMs with surface lubrication functionality were prepared. By converting the sliding friction of cartilage surfaces into rolling friction, this approach significantly reduced friction‐related damage to cartilage [165, 166]. Subsequently, researchers developed lubricated microspheres by dip‐coating the surface of GelMA HMs with a self‐adhesive polymer. They encapsulated the anti‐inflammatory drug diclofenac sodium (DS) to achieve dual functional performance aimed at alleviating the progression of OA [127] (Figure 4C). By anchoring HMs with liposomes and slowly releasing KGN, a dual effect of lubrication and treatment for OA was achieved [12]. Research has demonstrated the development of HMs with dual functions of drug release and lubrication for the treatment of OA, achieved through a dynamic Schiff base crosslinking network between carboxymethyl CS and oxidized dextran nanoparticles [167]. Preparing zwitterionic HMs that contain a high concentration of sulfonic betaine and carboxyl groups enhanced lubrication through hydration, and metformin was loaded to combat chondrocyte senescence [168].

To further enhance lubrication efficiency, HMs with dynamic cross‐linking were developed to load liposomes [169]. These microspheres enabled controlled release of liposomes under shear stimulation, improving lubrication efficiency. Current biological lubricants lack precise targeting of localized inflammatory lesions, and targeting the affected areas could further enhance lubrication efficiency [170]. A temperature‐responsive HM system based on HA was designed to achieve intelligent release of both drugs and lubricants for OA treatment [171]. However, studies have shown that the lubrication layer on the surface of HMs tends to delaminate and fail after repeated friction against cartilage. Therefore, efforts have been directed toward improving HMs with sustained lubrication functionality. These microspheres utilize an internal liposome reservoir to establish self‐renewing lubrication layers [172]. By incorporating cartilage CAPs loaded with FGF18‐targeting gene editing tools into hybrid exosomes, where membrane fusion hybridization integrates hybrid exosomes with external hydrophilic or lipophilic agents to form a self‐renewable hydrated layer, these were encapsulated in methacrylic anhydride‐modified HAMA HMs [161]. This resulted in the formation of an injectable microgel system with a self‐renewing hydrated layer, capable of providing sustained lubrication functionality.

### 4.4 Diagnostic and Imaging Applications

Fluorescent HMs show great promise in diagnosis and imaging. They can be fabricated by combining fluorescent materials (e.g., fluorescent dyes and quantum dots) with hydrogel matrices to produce microspheres with inherent fluorescence. When these microspheres interact with target substances (e.g., biomarkers, drug residues), the fluorescent signal changes. By detecting fluorescence intensity, color, or spectral characteristics, qualitative or quantitative analysis of target substances can be achieved. In food safety testing, Eu^3^⁺‐ or Al^3^⁺‐doped SA/polyvinyl alcohol HMs have been prepared, based on their distinct fluorescent responses to tetracycline and other antibiotics [173]. These fluorescent HMs can recognize tetracycline, as well as adsorb and remove it. Multicompartment microspheres used as microsensors have been fabricated with a spatially segregated hydrogel framework design to load analyte probes. When combined with the hybridization chain reaction, they enabled fluorescent signal amplification and detection of three mycotoxins (patulin, aflatoxin B1, and ochratoxin A) at the single‐particle level [174]. Such fluorescent HMs show great potential in food safety testing.

Fluorescent HMs also play a significant role in disease diagnosis. They have been used to detect cardiac troponin I and heart‐type fatty acid‐binding protein [175]. When combined with a smartphone as a detector, this approach enables label‐free chemiluminescent imaging immunoassays with a detection limit as low as the picogram‐per‐milliliter level. HMs encapsulating single cells have enabled the quantitative analysis of multiple microRNAs within individual cells, aiding in the differentiation of liver cancer cell subtypes [128] (Figure 4D).

## 5 Applications of HMs in Tissue System Diseases

HMs have emerged as versatile tools in biomedical applications, particularly for the treatment and management of various tissue system diseases. These polymer‐based structures possess unique properties, such as biocompatibility, controlled release, and tunable mechanical characteristics, that make them ideal for drug delivery, tissue engineering, and regenerative medicine. By encapsulating therapeutic agents, HMs can enhance localized treatment efficacy while minimizing systemic side effects. Furthermore, their ability to mimic the ECM provides a supportive environment for cell proliferation and tissue repair. This review explores recent advances in the use of HMs across different tissue systems, emphasizing their potential for translational applications.

### 5.1 Musculoskeletal System Diseases

MSDs are a group of conditions affecting bones, joints, muscles, ligaments, and associated structures. These conditions impair physiological function, significantly reduce quality of life, and pose a major global public health challenge. According to the World Health Organization, MSDs are among the leading causes of disability, particularly in aging populations. MSDs encompass joint diseases (e.g., OA and rheumatoid arthritis), muscle and ligament disorders (e.g., tendon rupture and tendinopathy), bone diseases (e.g., osteoporosis, fractures, and periodontal bone defects), and spinal disorders (e.g., intervertebral disc herniation). They can arise from genetic predisposition, environmental influences, mechanical stress, and inflammatory responses. The pathogenesis of MSDs is complex, involving multiple biological processes, including inflammatory mediators, metabolic abnormalities, and altered biomechanical loading. As understanding of these mechanisms advances, novel therapeutic strategies are emerging. HMs, as a new class of biomaterials, are gaining increasing attention. Studies have shown that HMs can effectively encapsulate drugs and achieve targeted release, thereby improving treatment of MSDs. Exploring the applications of HMs in MSD therapy therefore holds significant scientific and clinical value.

#### 5.1.1 Application of Cytokine‐Loaded HMs in Musculoskeletal Diseases

The use of HMs loaded with growth factors for the treatment of osteochondral injuries is among the earliest and most widespread applications [53, 176]. For example, gelatin‐based HMs releasing growth factors such as BMP‐2 and TGF‐β1 have been used to promote bone repair [177, 178]. Compared with single‐factor repair, the delivery of multiple growth factors is more effective in achieving composite repair. Dual delivery of VEGF and BMP‐2 via gelatin‐based HMs significantly enhanced healing in rat calvarial [179] and femoral defect repair [180]. Sequentially releasing TGF‐β3 and bFGF from HMs effectively promoted alveolar bone defect repair in rats [181]. Cai et al. [182] developed oppositely charged CS particles through a batch emulsion method to separately deliver bone‐inducing factors (BMP‐2) and antibacterial agents, thereby sustaining bone regeneration.

Controlled and sustained release of cartilage‐inducing factors such as TGF‐β1, TGF‐β3, and FGF‐2 from HMs has been widely applied in cartilage tissue engineering [183, 184, 185, 186, 187, 188, 189, 190, 191]. Using a double emulsion solvent extraction technique (w/o/w) to encapsulate TGF‐β3 and growth factor‐releasing peptides into microspheres promoted cartilage regeneration [192]. Chemokine‐based strategies to recruit autologous stem cells have also attracted significant attention. For instance, a HM loaded with TGF‐β1 liposomes recruited macrophages and BMSCs, promoted the chondrogenic differentiation of BMSCs, and alleviated OA progression [193]. Sustained release of platelet‐derived growth factor (PDGF)‐BB recruited endogenous MSCs, extending the paracrine activity of GelMA HMs for OA treatment [194]. Additionally, codelivery of PDGF‐BB and TGF‐β3 recruited autologous stem cells and promoted cartilage repair [195].

The clinical treatment of intervertebral disc degeneration (IVDD) remains challenging. Incorporating IL‐1ra, a natural antagonist of IL‐1β, into chondroitin sulfate‐functionalized microspheres suppressed hyperactive inflammation and restored the ECM in the nucleus pulposus (NP), providing an effective strategy for alleviating IVDD [196]. PDGF‐BB loaded onto Fn‐GelMA microspheres via the heparin‐binding domain of Fn recruited endogenous cells and promoted intervertebral disc repair [197].

**FIGURE 6:**
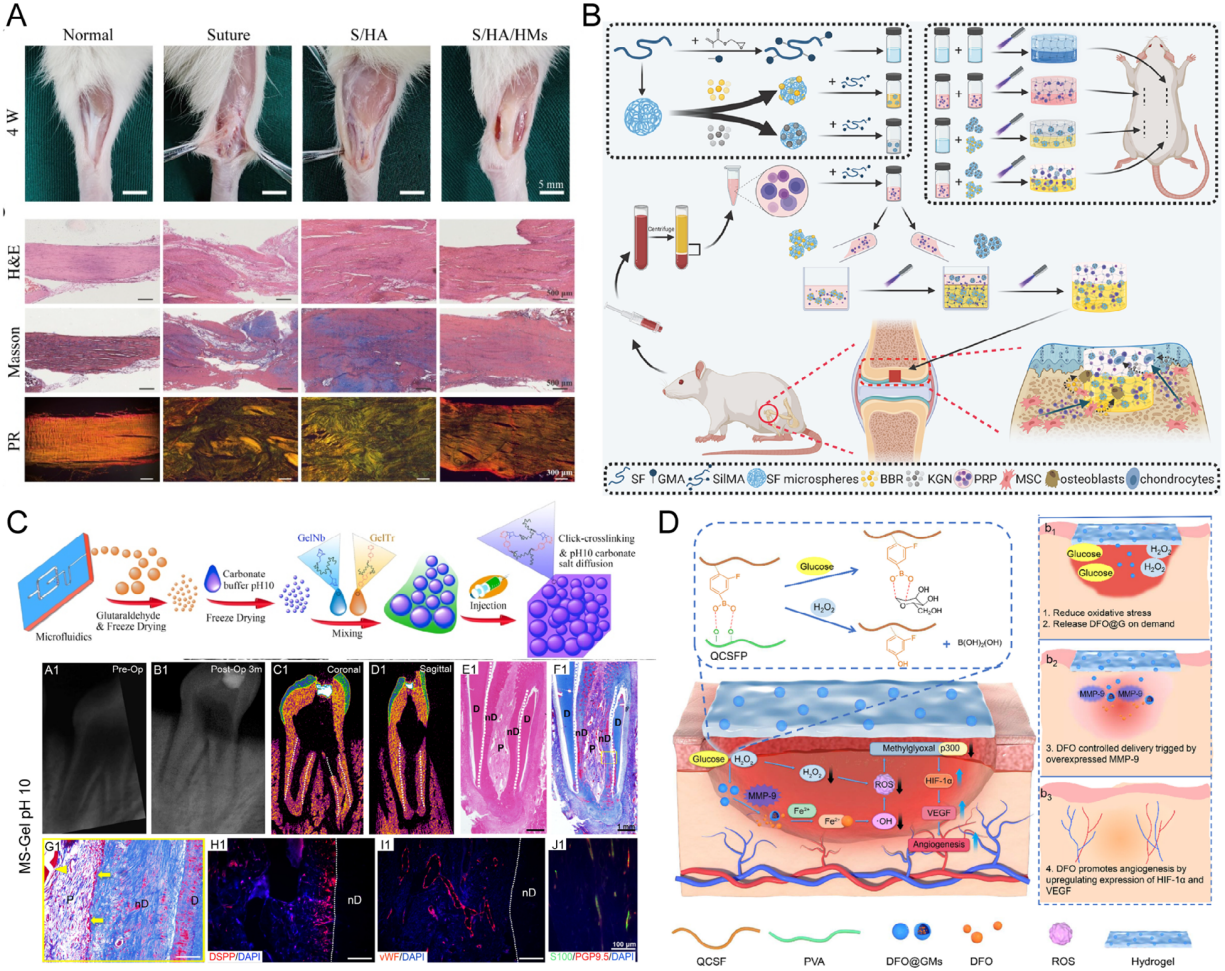
HMs applications in tissue system diseases. (A) Injectable composite microspheres/hydrogel membranes for Achilles tendon regeneration. Reproduced with permission [144]. Copyright 2025, The authors. (B) An all‐silk‐derived bilayer hydrogel for osteochondral tissue engineering. Reproduced with permission [207]. Copyright 2022, The authors. (C) Tissue repair by pH 10 MS‐Gel activated endogenous TGFβ1. Reproduced with permission [241]. Copyright 2022, The authors. (D) The fabrication and application of self‐adaptive DFO@G‐QCSFP for accelerating diabetic wound healing on the full‐thickness diabetic wound of a diabetic SD rat. Reproduced with permission [250]. Copyright 2023, The authors.

GelMA‐based HMs loaded with PDGF‐BB also promoted the migration and recruitment of TDSCs. When combined with an antiadhesive hydrogel membrane, these HMs facilitated high‐quality regeneration of ruptured Achilles tendons [144] (Figure 6A). Encapsulation of FGF19 in GelMA HMs enabled its sustained release in conjunction with MSCs, promoting myocyte recruitment and differentiation, facilitating myofibril growth, and significantly enhancing angiogenesis and skeletal muscle regeneration [198].

#### 5.1.2 Application of Small Molecule Drug‐Loaded HMs in Musculoskeletal Diseases

KGN is a small‐molecule drug used in cartilage regeneration [146]. However, its hydrophobicity results in uneven distribution and low bioavailability. Numerous studies have sought to improve its delivery. In a rat OA model, CS HMs capable of slowly releasing KGN into the joint cavity significantly slowed articular cartilage degeneration [199]. Emulsification‐prepared HMs loaded with KGN promoted MSC differentiation and improved OA progression [12, 13]. Ji et al. [200] developed CS‐based HMs incorporating KGN for sustained drug release, which promoted cartilage regeneration.

Anti‐inflammatory and antioxidant properties are also crucial for slowing OA progression. HMs modified with arbutin prevented OA advancement by enhancing local anti‐inflammatory and antioxidant responses [201]. Functionalized chondroitin sulfate HMs containing drug‐loaded liposomes delivered liquiritin to inhibit ROS damage, prevent polarization of transplanted macrophages to the M1 phenotype, and promote cartilage repair [202]. Vanillin, a natural anti‐inflammatory and antioxidant compound, was incorporated into multifunctional GelMA microspheres for localized delivery in intervertebral disc injury treatment [203].

Mitochondrial dysfunction is closely linked to ROS imbalance. Dual‐responsive HMs loaded with the Sirtuin 3 agonist dihydromyricetin restored mitophagy balance and promoted functional recovery after posttraumatic OA [204]. Similarly, HMs simultaneously loaded with the mitochondrial autophagy agonist urolithin A and the CAP WYRGRL enhanced mitochondrial autophagy, restored mitochondrial function, eliminated ROS, and maintained chondrocyte homeostasis [205].

Environment‐responsive biomaterials can improve drug efficacy. For example, bilayer (chondroitin sulfate/gelatin) HMs for sustained celecoxib release alleviated OA progression [149]. MMP‐13‐responsive HAMA HMs enabled sustained release of hydroxychloroquine, effectively eliminated ROS, and prevented cartilage degradation [150]. Multiple‐drug delivery strategies also demonstrated superior efficacy. CS‐based nanocomposite HMs coloaded with KGN and diclofenac exhibited a shear modulus comparable to cartilage and dual functions of promoting chondrogenesis and reducing inflammation [206]. Methyl methacrylate silk fibroin HMs effectively loaded KGN and berberine, achieving satisfactory regeneration of cartilage and subchondral bone [207] (Figure 6B).

Recruiting stem cells in situ with acellular scaffolds is an emerging strategy for tissue repair. Sequential release of Apt19s and KGN enabled spatiotemporal regulation of endogenous MSC recruitment and chondrogenic differentiation [208]. However, sustained‐release drugs alone are insufficient. Because of the dense structure and strong negative electrostatic shielding of articular cartilage, drugs have difficulty penetrating and diffusing to cartilage cells. To overcome this, a positively charged HM system was designed to exploit electrostatic attraction with the negatively charged cartilage matrix. This system penetrated deeply into the matrix, enabling drugs to target deeper cartilage cells [209, 210, 211].

Insufficient vascularization is a significant challenge in repairing severe bone defects. Deferoxamine (DFO) plays an important role in promoting angiogenesis, particularly for H‐type vessels, a specific vascular subtype that couples angiogenesis and osteogenesis. Injectable HMs loaded with DFO were used to treat large femoral defects in rats [212, 213]. Metal ions and trace elements can also serve as drugs to promote tissue repair [214]. For example, cerium ions (Ce^3^⁺) combined with phosphorylated HMs enhanced osteogenesis and angiogenesis [215].

#### 5.1.3 Application of Cell‐Loaded HMs in Musculoskeletal Diseases

The delivery of MSCs using HMs has garnered significant attention in treating skeletal system diseases. For instance, embedding bone marrow MSCs (BMMSCs)/BMP‐2 within GelMA HMs using a microfluidic device facilitated the repair of femoral defects in New Zealand white rabbits [216]. BMMSCs were also encapsulated in CS–collagen HMs and delivered to calvarial defects in mice, which similarly enhanced bone defect repair [217, 218]. A mechanically enhanced and bioactive SA composite HM effectively treated femoral defects in rats by encapsulating BMMSCs [219]. Furthermore, regulating the shape and pore size of HMs effectively promoted cell adhesion and proliferation, enhancing the osteogenic differentiation of BMMSCs in osteogenic induction medium [220].

Recently, researchers have shown growing interest in using electrical stimulation to enhance tissue repair. By applying wireless electrical stimulation to a novel conductive hydrogel loaded with dental pulp stem cells (DPSCs), this approach improved the paracrine effects mediated by DPSCs, thereby promoting regeneration of maxillary bone defects [221].

The use of HMs for cell delivery has also been extensively studied in cartilage tissue repair. Compared with conventional bulk hydrogels, PEG‐based HMs produced through microfluidic techniques effectively promoted cartilage matrix synthesis by human BMMSCs, likely due to particle microporosity [222, 223]. HA‐based HMs loaded with MSCs slowed OA progression [14]. Artificial cartilage particles produced by loading chondrocytes/MSCs into HMs effectively promoted cartilage defect repair [224, 225].

Inflammation‐related cells, such as macrophages, play a significant role in tissue repair. Precisely regulating macrophage activation is crucial for treating chronic inflammatory diseases such as OA. Inspired by innate immunity, researchers constructed HA/chondroitin sulfate HMs using microfluidic methods to recruit, capture, and reprogram pro‐inflammatory macrophages in the joint cavity, aiming to improve the inflammatory microenvironment in OA [226].

Cells maintained high viability within the HMs, which minimized apoptosis while preserving the genetic phenotype of annulus fibrosus cells, thereby facilitating intervertebral disc repair [227]. Excessive local inflammation in degenerative diseases can impair the effectiveness of biomaterial implants. To address this, a “peptide–cell–hydrogel” GelMA microsphere was developed by covalently coupling with the active peptide APETx2 and carrying NP cells. This approach inhibited local inflammation, regulated ECM metabolic balance, and alleviated IVDD [228].

Effective blood perfusion and skeletal muscle regeneration are crucial for treating ischemic limbs. Microfluidic technology was used to precisely transplant high doses of adipose‐derived MSCs (AD‐MSCs) into defect sites, enabling effective regeneration of ischemic limbs [198]. The incorporation of HMs into AD‐MSCs has also been shown to promote the healing and repair of ruptured tendons [229].

#### 5.1.4 Application of Nanoparticle‐Loaded HMs in Musculoskeletal Diseases

Due to their excellent magnetic responsiveness and controllability, magnetic nanoparticles are frequently incorporated into HMs, endowing them with additional functionalities. For example, gelatin microspheres were prepared using an emulsion crosslinking method and coated with PDA. Subsequently, amino‐modified magnetic nanoparticles (Fe_2_O_3_) and antibacterial nanoparticles were chemically modified onto these PDA‐coated hydrogels using a nanoprecipitation–self‐assembly approach, targeting the repair of periodontal bone defects [230].

Nanoparticles such as hydroxyapatite effectively reduced swelling and degradation rates of alginate‐based HMs, enhanced compressive strength, and significantly promoted osteogenic differentiation [42]. The dual incorporation of hydroxyapatite and gelatin HMs demonstrated a beneficial effect on osteoblast activity [231]. Injectable HMs functionalized with bisphosphonates were constructed through coordination reactions with metal ion ligands, allowing the capture of Mg^2^⁺ to promote reconstruction of osteoporotic bone defects [232].

In addition, nanoparticles addressed the challenge of diffusion within dense tissues in HMs. By constructing highly permeable selenium‐containing HMs, researchers eliminated ROS while inhibiting apoptosis of early chondrocytes, thereby restoring cartilage metabolic homeostasis [233]. Using microfluidic technology, zinc ion self‐assembled nanoparticles loaded with anti‐inflammatory drugs were immobilized in HMs. These nanoparticles and drugs were released into chondrocytes through a pH‐responsive mechanism, demonstrating effective anti‐inflammatory and antioxidant effects [234]. Yu et al. [151] developed cartilage‐targeting HMs with ROS responsiveness. By integrating cartilage‐targeting peptides and ROS‐responsive nanoparticles into HMs, they effectively alleviated OA progression.

Sulfhydryl polyhedral oligomeric silsesquioxane (POSS‐SH) was utilized as a nanostructured platform and encapsulated in HAMA HMs through microfluidic technology. An electromagnetic force was applied to release the positively charged drug encapsulated within the microspheres into deeper cartilage, alleviating OA progression [209]. POSS was also employed as a pH‐responsive nanogel composite system to prevent postmenopausal osteoporosis through oral administration and intestinal controlled release [235].

Sustained severe inflammation serves as the pathological basis for IVDD. Reducing local inflammatory responses helps to modulate the IVDD microenvironment and remodel the ECM of intervertebral disc cells. Using cell membrane encapsulation technology, a neutrophil membrane‐coated nanoparticle composite HM system loaded with TGF‐β1 was constructed. This system exhibited immunomodulatory effects and effectively improved the structure and biomechanical function of the NP [236]. Combining microfluidic and surface modification technologies, CS nanoparticles loaded with highly reductive BPQDs were grafted onto GelMA HMs. This approach alleviated extracellular acidosis in NP cells, blocked inflammatory cascade responses, and remodeled the ECM of intervertebral disc cells [237]. Limited nutrient availability and the accumulation of lactic acid significantly affect the survival and regenerative capacity of implanted cells. By chemically bonding MnO_2_ and lactate oxidase (LOX) composite nanoperoxidase to microfluidic HAMA microspheres, a locally injectable microsphere system for lactic acid depletion was developed. This system effectively enhanced the regeneration and repair of degenerated intervertebral discs in ischemic tissues [43]. By immersing LOX–MnO_2_ nanoperoxidase in decellularized NP HMs enriched with glucose, treatment for IVDD was achieved through nutrient provision and lactic acid depletion [238]. Research also developed a composite system of nanoparticle–HMs composed of TGF‐β and catalase to treat IVDD. This system worked by capturing hydrogen ions to block the NLRP3 cascade in disc inflammation [239]. IVDD could also be treated using HMs modified with active peptide nanoparticles [228]. MMP‐2 HMs encapsulating Smad3–siRNA nanoparticles were able to inhibit fibroblast proliferation and, in combination with self‐healing HA hydrogel, effectively prevent adhesions around the Achilles tendon [240].

HMs with lubricating functionality also play a crucial role in MSDs, as discussed in detail in Section 4.3.2. This topic will not be further elaborated in the current section.

### 5.2 Neurological Diseases

HMs also play a positive therapeutic role in the field of neural repair. An alkaline HM delivering TGFβ‐1 effectively promoted the regeneration of dental pulp nerves and dentin [241] (Figure 6C). Encapsulating human DPSCs in GelMA HMs effectively promoted dental pulp nerve regeneration [242]. Bionic HMs loaded with insulin effectively promoted the regeneration of axons and myelin sheaths in injured sciatic nerves [243]. Supramolecular HMs of PDGF‐mimicking peptides, prepared through electrospinning, sustained the proliferation of neural stem cells and inhibited cell apoptosis, exerting powerful neuroprotective effects and promoting recovery from spinal cord injuries [244]. An unfavorable microenvironment significantly hinders the recovery of spinal cord injuries. However, by loading MG53 into HMs combined with a nerve scaffold, the system was able to respond to MMP proteins and provide stable release of MG53. This approach effectively improved motor function in mice, suppressed neuroinflammation, and promoted nerve and synaptic regeneration [245].

HMs also offer a promising therapeutic strategy for long‐term postoperative pain management. Peripheral nerve block can precisely control pain while reducing the side effects of opioids. Using microfluidic techniques to prepare ropivacaine‐loaded HMs enabled prolonged retention and sustained drug release in vivo, with sensory and motor block durations of 48 and 36 h, respectively [246]. HMs encapsulating bupivacaine and dexmedetomidine also provided effective and prolonged analgesia over time [247].

### 5.3 Dermatological Diseases

In the field of skin tissue engineering, designing smart structural color HMs not only promoted wound angiogenesis through the release of VEGF but also enabled effective monitoring of the growth factor release process using the unique visual color changes of the hydrogel [248]. In addition, HMs loaded with VEGF and natural bioactive components such as microalgae effectively promoted wound healing [249]. In the field of accelerating chronic wound healing, gelatin microspheres releasing DFO promoted angiogenesis, thereby enhancing their potential for treating diabetic wounds [250] (Figure 6D). Researchers also found that bacteria could serve as therapeutic agents to accelerate the healing of infected wounds. They prepared HMs encapsulating beneficial bacteria (*Lactobacillus rhamnosus*) using an emulsion method. These beneficial bacteria promoted wound closure and new tissue regeneration through their secretion of antimicrobial substances and anti‐inflammatory properties [251].

In addition, metal ions can serve as special agents to accelerate chronic wound healing by regulating the pH of the wound, affecting the proliferation and migration of surrounding cells, and modulating the activity of various biological factors in the wound. For example, CS‐based HMs loaded with magnesium ions could release or absorb hydrogen ions to enhance the healing of chronic wounds [252]. The sustained release of copper ions achieved excellent antibacterial properties in the skin and promoted wound healing [253]. Zinc ions, as an important trace element, can also exert antibacterial and anti‐inflammatory effects. When loaded in HMs, they significantly promoted wound healing [254]. Inspired by the natural organic/inorganic adhesives secreted by oysters, Zn^2^⁺‐functionalized nanocomposite microgels can self‐assemble into highly stretchable and adhesive fused granular hydrogels. These hydrogels can in situ fill irregularly shaped wounds, significantly accelerating healing in various wound models [255].

Excessive cell‐free DNA (cfDNA) can induce chronic inflammation by activating intracellular nucleic acid sensors. HMs loaded with PDA nanosheets effectively and specifically reduced cfDNA concentrations in the pathological microenvironment. Additionally, the catechol in PDA nanosheets helped lower ROS levels and significantly promoted healing in a diabetic wound mouse model [256]. Recent research has clarified that excessive release of neutrophil extracellular traps (NETs) is a central factor in the pathogenesis of inflammation dysregulation during diabetic wound healing, significantly contributing to delayed healing. By developing a novel bio‐based HM for NETs clearance, it was possible to noninvasively reduce the pro‐inflammatory responses associated with diabetic wounds [257].

### 5.4 Cardiovascular and Respiratory System Diseases

HMs also demonstrate therapeutic efficacy in cardiac repair. They can be precisely delivered to damaged areas using a minimally invasive catheter‐based strategy [258]. In a rat myocardial infarction model, the sustained delivery of FGF from gelatin microspheres promoted angiogenesis and improved ventricular function [259, 260, 261]. Alginate HM patches loaded with angiogenic growth factors were used for the local delivery of VEGF to ischemic heart tissue [262]. PEG/poly(butylene terephthalate) HMs loaded with VEGF demonstrated therapeutic effects in the treatment of myocardial infarction [263]. Low circulating levels of insulin‐like growth factor 1 (IGF‐1) have been associated with an increased risk of cardiovascular diseases. The preparation of silk fibroin‐based HMs for the sustained release of IGF‐1 was shown to effectively treat myocardial infarction and improve ventricular function [264]. Furthermore, HA‐based HMs loaded with IL‐10 [265], prepared using microfluidic chip technology, also demonstrated efficacy in promoting cardiac repair when delivered through shear‐thinned supramolecular hydrogels.

Endothelial cell transplantation is a promising approach in vascular tissue engineering. However, this therapy is hindered by significant cell apoptosis upon transplantation and poor recruitment of host wall cells to stabilize the newly formed blood vessels. HMs that deliver both VEGF and monocyte chemoattractant protein‐1 significantly enhanced endothelial cell survival and promoted functional vessel formation [266]. HMs encapsulating VEGF‐overexpressing HEK293T cells effectively promoted angiogenesis [267]. Compared with single‐cell types, the coencapsulation of human BMMSCs and human umbilical vein endothelial cells in GelMA HMs more effectively promoted angiogenesis [268]. HMs loaded with angiogenic growth factors also promoted vascularization, leading to the regeneration of dental pulp‐like tissue [269].

HMs are widely used for drug delivery in the respiratory system, as they are well‐suited for creating particles of an appropriate size for respiratory administration (∼5 µm) [270]. Conventional bulk hydrogels are not suitable for pulmonary drug delivery, as they pose a risk of airway obstruction. Early studies by Hussain et al. [271] found that embedding antituberculosis drugs in HMs increased their bioavailability in the respiratory tract by nine times and effectively shortened the duration of treatment [272]. Through microsphere encapsulation, the half‐life and mean residence time of the antituberculosis drugs increased by 13 to 15 times [273]. To ensure continuous drug delivery to the lungs, aerosol delivery is typically employed; therefore, particles with a diameter of 0.5–5 µm are needed to penetrate the deeper regions of the lungs [274]. After the dry drug‐loaded particles enter the respiratory tract, they absorb water and expand upon depositing on the moist lung surfaces [275]. Drugs must have a size greater than 5 µm to avoid premature uptake and clearance by alveolar macrophages [270]. Additionally, by using mucoadhesive hydrogel particles for sustained therapeutic delivery in the respiratory tract, the drug clearance rate can also be reduced [276]. MMPs (such as MMP‐2) are overexpressed in many pulmonary diseases. By designing protease‐responsive degradable HMs, it is possible to treat a range of conditions, including lung cancer, tuberculosis, and chronic obstructive pulmonary disease [277, 278].

### 5.5 Digestive System Diseases

Ulcerative colitis (UC), classified as an inflammatory bowel disease (IBD), is a common inflammatory condition affecting the gastrointestinal tract. Oral anti‐inflammatory medications are the first‐line and widely accepted clinical treatment. However, oral medications for UC often face complex challenges, such as insufficient drug accumulation, limited mucosal barrier permeability, and difficulties in clearing excessive ROS and inflammatory cytokines. By designing SA HMs loaded with M2 macrophage membrane‐coated nanomotors, targeted therapy for UC was made possible [279]. Utilizing the characteristics of calcium alginate, which is insoluble in the stomach and soluble in the intestine, the microspheres could accurately “burst” at the colonic site after oral administration. The microspheres adhered to the intestinal wall, locally regulated macrophage polarization, and induced a beneficial gut microbiome composition to treat IBD [280]. Alginate HMs loaded with indomethacin demonstrated pH‐sensitive properties and were used for targeted drug delivery to the small intestine [281]. Composite HMs loaded with curcumin also enabled targeted drug delivery to the small intestine [282, 283].

Modulating the gut microbiota is also an attractive strategy. Pectin–alginate HMs encapsulating the probiotic *Lactobacillus kefiranofaciens* reduced gastric acid erosion, promoted targeted delivery to the colon, and facilitated the sustained release of probiotics [284]. HMs encapsulating *Bifidobacterium* and drug‐modified dietary fibers protected the drug from acidic and enzymatic degradation, delivering it to the colon for IBD treatment [285]. *L. rhamnosus* was also shown to significantly enhance intestinal barrier function and remodel the gut microbiota [286]. Selenoproteins play a crucial role in immune cells and the regulation of inflammation. However, selenoproteins easily denature or degrade in acidic gastric environments. Inspired by the acid resistance of calcium alginate hydrogel, encapsulating selenoproteins effectively improved their stability and reduced inflammation associated with IBD [287].

Many drugs, such as DS, can cause strong gastric irritation. However, through the use of alginate HMs combined with nanoparticles, a slow‐release formulation of DS was achieved. Pharmacokinetic studies in vivo indicated that the composite HMs provided improved drug bioavailability. The liver, as a key detoxification organ, inspired the development of biomimetic enzyme cascade reaction HMs, designed to replicate the alcohol detoxification function of a liver‐on‐a‐chip system [288]. HMs can also be used to construct in vitro organoid models for drug testing. Alginate‐based HMs loaded with hepatocytes were designed to mimic a biomimetic endothelial liver barrier, serving as a platform for drug testing [289]. The lack of stable hepatocyte sources remains one of the major limitations in the clinical application of stem cell transplantation and bioartificial livers. Alginate‐based HMs were used to differentiate human embryonic stem cells into hepatocyte‐like cells, offering a potential therapeutic approach for clinical use [290].

Diabetes, as a prevalent disease, has garnered significant attention. By coencapsulating pancreatic islet cells and MSCs in alginate‐based HMs and implanting them intraperitoneally in a diabetic mouse model, blood glucose levels and intraperitoneal glucose tolerance tests were monitored. The results showed that, compared with the group receiving islet cell transplantation alone, the islet cell/MSC/HM group exhibited a significant improvement in blood glucose response [291]. This suggests that MSCs and HMs can substantially enhance the survival and function of pancreatic islet cells.

### 5.6 Current Status of Clinical Translation

HMs, characterized by their exceptional biocompatibility and customizable drug release profiles, have emerged as valuable tools in clinical applications requiring localized and sustained drug delivery or tissue augmentation. In a 2025 multicenter, randomized, double‐blind, parallel‐grouped, positive‐controlled, noninferiority trial, Pan et al. [292] investigated the efficacy and safety of Aphranel, an injectable calcium hydroxylapatite microsphere hydrogel filler, versus Restylane for correcting nasolabial folds in Chinese subjects. Their findings demonstrated that a single injection of Aphranel prolonged dermal residence time. Notably, the investigator‐assessed Wrinkle Severity Rating Scale improvement rate at 24 weeks reached 84.04% for Aphranel, compared with 78.72% for Restylane, confirming noninferior efficacy. Additionally, the safety profile was favorable, with adverse events (e.g., injection site swelling and pain) occurring at a comparable rate to the control group.

In the realm of ischemic disease treatment, Marui et al. [293] reported on a phase I–IIa study in 2007 utilizing biodegradable gelatin HMs loaded with bFGF to induce therapeutic angiogenesis in patients with critical limb ischemia (CLI). Intramuscular administration of these microspheres yielded significant improvements: the 6 min walking distance increased from 295 ± 42 to 491 ± 85 m at 24 weeks, transcutaneous oxygen pressure (TcO_2_) rose from 53.5 ± 5.2 to 65.5 ± 4.0 mmHg, and rest pain scores decreased from 3.5 ± 0.2 to 1.0 ± 0.6. These findings were further corroborated in a subsequent phase I–IIa study by Kumagai et al. [294], which demonstrated significant improvements in TcO_2_ (from 28.4 ± 8.4 to 46.2 ± 13.0 mmHg at 24 weeks, *p* < 0.01) and Rutherford classification in patients with CLI, with no serious treatment‐related adverse events reported. Similarly, a 2009 phase I trial by Hashimoto et al. [295] evaluated the selective and sustained delivery of bFGF using acidic gelatin HMs for peripheral arterial disease. This study confirmed good tolerability without severe complications, with all patients experiencing symptomatic improvements.

Current clinical investigations of HMs are largely confined to soft tissue augmentation and ischemic disease treatment (Table 3), reflecting constraints imposed by material properties (batch‐to‐batch variability) and the complexity of clinical indications. Beyond standard ethical considerations in clinical research, unique challenges specific to HMs as biomaterials demand attention: these include the long‐term effects of degradation products on tissues, potential immunogenicity from crosslinking agents, and the need for transparent informed consent regarding material‐related uncertainties. Additionally, a set of ongoing clinical trials has been compiled, with further details available in Table 3. Moving forward, advancements in material engineering and expanded clinical indications will necessitate more rigorously designed trials to facilitate the broader clinical translation of HMs.

**TABLE 3:** Current clinical trials of HMs.

HMs	Study type/phase	Author/conducting institution and year	Diseases	Methods/key trial details
Injectable calcium hydroxylapatite microsphere hydrogel fillers	Multicenter, randomized, double‐blind, parallel‐grouped, positive‐controlled, noninferiority clinical trial	Pan et al. [292]	Nasolabial fold correction	210 subjects were randomized to receive bilateral nasolabial fold treatment with Aphranel and Restylane. The primary efficacy endpoint was the WSRS improvement rate at 24 weeks.
Biodegradable gelatin HMs loaded with bFGF	Phase I–IIa clinical study	Marui et al. [293]	Critical limb ischemia	7 CLI patients received intramuscular injection of 200 µg bFGF‐incorporated gelatin HMs into the gastrocnemius of the ischemic limb. Follow‐up for 24 weeks.
Biodegradable gelatin HMs loaded with bFGF	Phase I–IIa clinical study	Kumagai et al. [294]	Critical limb ischemia	10 CLI patients received a single intramuscular injection of 200 µg bFGF‐incorporated gelatin HMs. Follow‐up for 24 weeks.
Acidic gelatin HMs loaded with bFGF	Phase I trial	Hashimoto et al. [295]	Peripheral arterial disease	8 PAD patients (eight limbs) received intra‐arterial infusion of AGHM suspension containing 100 µg bFGF into the affected limb. Follow‐up for ≥6 months.
Cross‐linked sodium hyaluronate gel containing hydroxyapatite microspheres	Not applicable	Sichuan University/2024–2027	Temporal depression	Trial number: ChiCTR2500107432; Status: recruiting
Poly‐l‐lactic acid sodium hyaluronate gel microspheres	Not applicable	Beijing Hospital/2024–2026	Micropenis syndrome	Trial number: ChiCTR2500095822; Status: recruiting
PaMZ/BMP‐2 thermosensitive gel microsphere‐controlled release complex	Phase 0	The Second Affiliated Hospital of Zunyi Medical University/2025	Spinal tuberculosis	Trial number: ChiCTR2400095016; Status: not yet recruiting
Gelatin microspheres (Nexsphere)/tri‐acryloyl gelatin microspheres	Not applicable	Severance Hospital 2021	Symptomatic uterine fibroids	Trial number: NCT05086770; Status: completed
Tretinoin gel microspheres (0.1%)	Early phase I	Catawba Research/2020‐2021	Acne vulgaris	Trial number: NCT04883736; Status: completed
Tretinoin (0.04%)–clindamycin (1%) composite gel microspheres	Phase III	Dr Reddys Laboratories/2014	Acne vulgaris	Trial number: CTRI/2014/08/004830; Status: completed
Tretinoin (0.1%)–clindamycin (1.2%) fixed–dose composite gel microspheres	Phase III	Glenmark Pharmaceuticals/2012	Acne vulgaris	Trial number: CTRI/2012/03/002472; Status: completed
Tretinoin gel microspheres (0.04%)	Phase III	Cu‐Tech 2009‐2014	Acne vulgaris	Trial number: NCT01243450; Status: completed
Tretinoin gel microspheres (0.1%)	Phase III	Moore Clinical Research 2009‐2014	Acne vulgaris	Trial number: NCT01135069; Status: completed
Tretinoin gel microspheres (0.08%)	Not applicable	Catawba Research India/2024	Acne vulgaris	Trial number: CTRI/2024/07/069994; Status: closed to recruitment
Tretinoin gel microspheres (0.08%)	Not applicable	Lets Evolve Life Pvt. Ltd/2023	Acne vulgaris	Trial number: CTRI/2023/04/052062; Status: open to recruitment
Tretinoin gel microspheres (0.06%)	Phase I	G7 Synergon Private Limited/2023	Acne vulgaris	Trial number: CTRI/2023/01/049053; Status: completed

*Data source*: ClinicalTrials.gov website (https://trialsearch.who.int/; https://www.chictr.org.cn/).

### 5.7 Potential for Cross‐System Application

HMs exhibit remarkable potential for cross‐system applications in tissue regeneration, leveraging their tunable structure and multicomponent loading capacity to coordinate multiple biological processes. In bone regeneration, porous GelMA‐based HMs achieve synergistic osteogenesis and angiogenesis by optimizing the coculture ratio of BMSCs and HUVECs, promoting paracrine effects that enhance both bone formation and vascular network development, which significantly accelerates vascularized bone repair in rat cranial defects [296]. For infected bone defects, acoustically responsive HMs integrate anti‐infection and osteogenesis: ultrasound‐triggered release of EGCG achieves 99% antibacterial efficiency against MRSA, while MoS_2_ nanoparticles promote osteogenic differentiation, reducing inflammation and enhancing new bone formation in a rat osteomyelitis model [297].

Beyond bone–vascular systems, core–shell microspheres with sequential release capabilities regulate neuro–vascular–bone crosstalk: cerium oxide nanoparticles mitigate acute inflammation by scavenging ROS, while spinal white matter ECM and NGF promote axonal regeneration and vascular ingrowth, improving motor function recovery in spinal cord‐injured rats [298]. Similarly, dual‐electroactive cryogel microspheres synchronously enhance osteogenesis, angiogenesis, lymphogenesis, and neurogenesis through piezoelectric/conductive properties and ion release, accelerating calvarial defect repair [299]. In soft tissue repair, organoid‐like neurovascular spheroids and dermal neurovascularized spheroids integrate neural, vascular, and skin cells, boosting skin flap survival and reconstructing dermal tissue via coordinated neurovascular regeneration [300]. These studies collectively demonstrate that HMs, through rational design, can orchestrate cross‐system interactions, offering innovative solutions for complex tissue repair scenarios.

## 6 Challenges and Limitations in Clinical Translation

Current research primarily focuses on the advantages of HMs in fields such as drug delivery and tissue repair, yet there is insufficient discussion regarding the core obstacles in their clinical translation. Despite the significant potential of HMs in regenerative medicine, their translation from laboratory to clinical practice is still confronted with multiple challenges in materials, manufacturing, immunological safety, and other aspects. This section systematically outlines these bottlenecks, including difficulties in large‐scale manufacturing, batch‐to‐batch variations, biocompatibility risks, insufficient mechanical properties, the complexity of multifunctional systems, and regulatory hurdles.

### 6.1 Challenges in Large‐Scale Manufacturing and Batch‐to‐Batch Variability

Current methods for preparing HMs still have significant limitations, facing multiple challenges in manufacturing scale‐up, large‐scale production, and control of batch‐to‐batch variations. At the technical level, different preparation methods each have their drawbacks: while microfluidic technology can produce monodisperse microspheres (with a size coefficient of variation of 1.3–3.9%) [64, 65, 66], its production yield is low, making it difficult to meet industrial‐scale demands. Electrospray technology relies on high‐voltage equipment, suffering from low droplet collection efficiency and poor morphological control. While batch emulsion technology offers high production yields, it results in a wide size distribution [73, 75] and poor uniformity. Potential solutions, such as air microfluidics (capable of increasing production yields by 10–100‐fold) [104, 105] and parallelized microfluidic chips (which enhance efficiency through multichannel designs), remain in the laboratory stage and have not yet been translated into industrial applications.

In terms of compliance, establishing standardized processes that meet GMP standards is crucial, with ensuring sterility being the core challenge. PDMS, a material commonly used in microfluidic chips, tends to adsorb microorganisms due to its porous structure and thus requires ethylene oxide treatment for sterilization. In batch emulsion processes, the mixing of oil and aqueous phases is prone to introducing environmental contaminants, necessitating full‐process control through 0.22 µm membrane sterile filtration and stirring within a closed sterile system, which increases equipment costs and operational complexity. For electrospray equipment, stable monitoring of parameter fluctuations such as voltage and flow rate is essential to avoid contamination risks caused by process instability.

Furthermore, controlling batch‐to‐batch variability poses an equally significant challenge. Sources of such variability include fluctuations in mechanical mixing intensity and emulsification time during the emulsion process, which lead to differences in droplet size and crosslinking degree [82]. Variations in the sources of natural polymers, such as compositional fluctuations among batches extracted from animal tissues, can affect microsphere performance. For synthetic materials, uneven molecular weight distribution leads to inconsistent degradation rates. Existing quality control methods (such as dynamic light scattering for particle size measurement) struggle to cover differences in biological functionalities, such as drug release kinetics and cell compatibility. To enhance uniformity, strategies are needed, including the introduction of AI‐assisted digital microfluidic systems [60] for real‐time parameter regulation, the establishment of material traceability systems (such as standards for molecular weight distribution of synthetic polymers), and the adoption of automated production equipment (such as piezo‐driven systems integrated with optical sorting).

### 6.2 Biocompatibility and Immunological Safety Risks

HMs with different compositions exhibit distinct degradation patterns in vivo. The degradation of natural polymers is highly dependent on the in vivo enzymatic environment [150], such as the specific degradation of gelatin by MMPs. However, variations in enzyme activity among individuals and local pathological conditions (such as elevated enzyme concentrations in inflammatory regions) may lead to significant fluctuations in degradation rates, thereby affecting functional stability [151]. Animal‐derived biopolymers exhibit compositional heterogeneity and batch‐to‐batch variations, which may lead to inconsistent performance. Additionally, they can induce immunogenicity either inherently or due to residual impurities [301]. Moreover, the gelation of biopolymers typically requires toxic reagents or photoinitiators, as exemplified by GelMA hydrogels, the most widely used biomaterial of this type.

It is worth noting that as the use of PEG in pharmaceuticals has increased, concerns regarding its immunogenicity have also emerged. Researchers have found that PEG may trigger the production of anti‐PEG antibodies in the body, potentially causing adverse immune reactions [39, 40]. Nanoparticles may also induce in vivo immune responses, inflammation, and hemolysis [302]. Consequently, it is necessary to control the dosage of nanoparticles and develop more biosafe materials.

### 6.3 Mechanical Durability and in Vivo Functional Stability

HMs face significant challenges in terms of mechanical durability and functional stability in vivo. A core issue lies primarily in the inadequate matching between their mechanical properties and the physiological environment. In dynamic environments such as the joint cavity, HMs must withstand long‐term repeated friction, making the lubricating layer of microspheres for cartilage repair highly susceptible to failure [53]. In bone tissue engineering, highly rigid microspheres designed to meet supportive requirements [52] may undergo brittle fracture due to stress concentration.

This mismatch in mechanical properties further triggers a cascade of functional failure mechanisms: mechanical degradation damages microspheres, causing premature release of loaded drugs (e.g., growth factors) and compromising controlled release efficacy. Concurrently, disruption of the crosslinked network results in the leakage of encapsulated cells, reducing cell viability. Therefore, it is essential to match HMs with varying mechanical properties to different biomechanical environments.

### 6.4 Complexity and Reproducibility Challenges of Multifunctional HMs

Multifunctional HMs face dual challenges of complexity and reproducibility in the process of integrating multiple functions to enhance their clinical application value. To achieve combined functionalities such as drug delivery, lubrication, and targeting, components with distinct properties need to be integrated into a single system [127]. However, these components may conflict due to differences in their chemical properties, thereby compromising the structural stability of the microspheres. The fabrication of complex configurations such as core–shell structures relies on cumbersome layered crosslinking steps [303]. During scale‐up production, structural defects often occur due to parameter fluctuations, reducing product reproducibility.

This complexity in functional integration also extends to the regulatory realm, when HMs exist as combination products, they are subject to more stringent regulatory requirements by authorities such as the US FDA. Not only is it necessary to verify the safety of individual components, but additional evidence must be provided to demonstrate that the synergistic effects of each functional module do not induce unintended toxicity. This undoubtedly increases the difficulty of clinical translation.

### 6.5 Barriers to Clinical Translation and Lack of Standardization

The clinical translation of HMs is hindered by translational barriers and the lack of standardization, delaying their progression from laboratory research to clinical application. A primary obstacle lies in the limitations of preclinical models: significant discrepancies exist between the microenvironments of animal models and the human body [304, 305]. As a result, therapeutic strategies that are effective in animal experiments often fail to replicate such success in human clinical trials. Furthermore, the industry lacks unified standards, with key indicators such as toxicity thresholds for degradation products and detection methods for immunogenicity remaining unstandardized. This not only impairs the comparability of research results but also poses significant challenges to safety assessments. Currently, most studies remain in the phase I–IIa stages [292, 293]. For instance, the research conducted by Kumagai et al. [294] on the treatment of CLI has demonstrated encouraging preliminary results, but it lacks large‐scale, multicenter phase III clinical trial data. This makes it difficult to comprehensively verify its long‐term safety and efficacy, thereby posing significant challenges to the clinical application and promotion of HMs.

## 7 Conclusions and Future Prospects

HMs have grown into a key platform in biomedical research, linking material science, tissue engineering, and clinical practice. Over recent decades, their evolution has moved beyond simple drug carriers to multifunctional systems. These systems can precisely deliver cells, bioactive factors, and nanoparticles, while mimicking the ECM to support tissue regeneration. The adaptability of HMs comes from their adjustable material properties, whether from natural polymers like gelatin and alginate or synthetic ones such as PEG, and advanced fabrication methods like microfluidics and EHD spraying. These allow control over size, shape, and degradation rates, making HMs ideal for minimally invasive therapies that address unmet needs in areas ranging from musculoskeletal repair to chronic wound healing.

The clinical value of HMs is becoming increasingly clear across various diseases. In MSDs, HMs loaded with chondrogenic drugs or MSCs have shown promise in reversing OA and repairing bone defects. They achieve this by combining lubrication, anti‐inflammatory effects, and regenerative stimulation. For ischemic diseases, gelatin‐based HMs releasing bFGF have promoted therapeutic angiogenesis, improving blood flow and function in CLI. In drug delivery, HMs have proven effective too, whether through pH‐responsive release for gastrointestinal disorders or targeted pulmonary delivery for tuberculosis, demonstrating their ability to overcome biological barriers and enhance drug effectiveness. These advances highlight the potential of HMs to bridge the gap between laboratory research and clinical applications.

Even with these successes, significant challenges stand in the way of fully translating HMs into clinical use. Large‐scale production still struggles with consistency between batches, especially with methods like batch emulsion, which limits their application in clinical settings. Biocompatibility issues, such as immune reactions from crosslinking agents or unpredictable breakdown products, necessitate comprehensive long‐term in vivo studies. Additionally, multifunctional systems that combine drug release, cell delivery, and environmental responsiveness face challenges in reproducibility and regulatory approval, as their combined functions require stricter safety verification. Overcoming these issues will require collaboration across disciplines, integrating material engineering with automation technologies such as AI‐assisted microfluidics and implementing standardized quality control processes.

The future of HMs will focus on smart and integrated designs. Stimuli‐responsive HMs, which activate in response to pathological signals such as inflammation or hypoxia, will enable drugs to be released when needed. This will maximize treatment effectiveness while reducing off‐target effects. Cross‐system applications, such as coordinating nerve and blood vessel regeneration in spinal cord injuries or linking bone formation with angiogenesis in bone repair, will leverage the modular nature of HMs to address complex tissue repair processes. Furthermore, combining HMs with emerging technologies such as 3D bioprinting and single‐cell analysis could lead to personalized treatments tailored to individual patients. As these innovations progress, HMs are poised to make a significant impact on regenerative medicine, offering minimally invasive, highly targeted therapies that restore tissue function and improve quality of life for patients worldwide.

## Author Contributions

M.Y., Y.S., F.W., J.C., H.H., and J.W. drafted the manuscript. M.Y., X.Z., C.Z., and Z.Z. prepared the figures. J.S., F.Y., H.S., J.H., P.L., and P.Y. edited and revised the manuscript. J.W., H.H., and J.C. approved the final version of manuscript. All authors have read and approved the final manuscript.

## Ethics Statement

The authors have nothing to report.

## Conflicts of Interest

The authors declare no conflicts of interest.

## Data Availability

The authors have nothing to report.
